# Supernatant of Water Extraction–Ethanol Precipitation of *Chaga mushroom* Improves Hyperglycemia in Type 2 Diabetes Mellitus and Is Accompanied by Changes in Gut Microbiota Composition

**DOI:** 10.3390/foods15132383

**Published:** 2026-07-03

**Authors:** Yang Yang, Xiaoxuan Du, Xiaodong Ge, Huangcong Ding, Xingwei Wang, Xiaoyu Gao, Gulnigar Adiljan, Yaxuan Wang, Junchao Chen, Ligen Chen, Xiaoqing Zhu, Wei Xu, Kyung-Min Kim, Rong Shao

**Affiliations:** 1 School of Chemistry & Chemical Engineering, Yancheng Institute of Technology, Yancheng 224051, China; 15189416922@163.com; 2 College of Marine and Bioengineering, Yancheng Institute of Technology, Yancheng 224051, China; 17895266026@163.com (X.D.); gexiaodongyg@163.com (X.G.); 15366364870@163.com (H.D.); ycsf105@163.com (X.W.); 18913064550@163.com (X.G.); 19705102832@163.com (G.A.); 19516306017@163.com (Y.W.); 18852641055@163.com (J.C.); ycit549638894@outlook.com (L.C.); xuweiyc@163.com (W.X.); 3 Coastal Agriculture Research Institute, Kyungpook National University, Daegu 41566, Republic of Korea; 4 Jiangsu Key Laboratory for Exploration and Utilization of Marine Wetland Biological Resources, Yancheng Institute of Technology, Yancheng 224051, China; 5 Canada Menghe Oriental Medicine Research Institute, Richmond, BC V6V 3E3, Canada; dr.x.zhu@hotmail.ca; 6 Department of Applied Biosciences, Kyungpook National University, Daegu 41566, Republic of Korea

**Keywords:** *Chaga mushroom*, type 2 diabetes mellitus, gut microbiota, hyperglycemia

## Abstract

Type 2 diabetes mellitus (T2DM) is a chronic metabolic disorder characterized by insulin resistance and inadequate insulin secretion. Prolonged hyperglycemia is associated with disruption of gut microbiota (GM) homeostasis. Although bioactive compounds in *Chaga mushroom*, such as polysaccharides, have hypoglycemic effects, research on its polysaccharide preparation by-product, the supernatant of water extraction–ethanol precipitation of *Chaga mushroom* (SWEPC), remains limited. In this study, we investigated the effects of SWEPC on GM composition and its therapeutic potential in alleviating hyperglycemia in T2DM mice. After four weeks of SWEPC intervention (300 mg/kg/day), T2DM mice exhibited significant improvements: fasting blood glucose (FBG) decreased by 52.29% (*p* < 0.001); the area under the curve of oral glucose tolerance decreased by 37.68% (*p* < 0.001); glycated serum protein decreased by 65.36% (*p* < 0.001); the insulin resistance index decreased by 44.42% (*p* < 0.001); and the insulin sensitivity index increased by 90.24% (*p* < 0.001). Spearman’s correlation analysis revealed that *Anaeroplasma*, *Anaerotruncus*, and *Ruminococcus* were associated with serum low-density lipoprotein cholesterol (r = 0.75), FBG (r = −0.81), and body weight (r = −0.65), respectively, suggesting their potential involvement in T2DM-related metabolic traits and warranting further investigation. Overall, SWEPC ameliorated hyperglycemic manifestations in T2DM mice, which was accompanied by changes in the GM community.

## 1. Introduction

Type 2 diabetes mellitus (T2DM) is a multifactorial disease influenced by genetic predisposition, epigenetic regulation, early-life programming, and environmental factors [1]. Among these factors, dietary patterns play a particularly prominent role. With the accelerated pace of modern life, the consumption of high-salt, high-sugar, and high-fat diets has increased substantially, contributing to the increasing prevalence of chronic metabolic disorders [2]. According to the most recent International Diabetes Federation Diabetes Atlas (11th edition, 2025), an estimated 589 million adults aged 20–79 years had diabetes in 2025, with projections predicting an increase to 853 million by 2050 [3]. The 11th edition also refined the classification of diabetes, formally recognizing malnutrition-related diabetes mellitus as type 5 diabetes mellitus, in addition to type 1 diabetes mellitus, T2DM, gestational diabetes, and other specific types [4]. T2DM remains the predominant form, accounting for over 90% of all diabetes cases [5]. Importantly, T2DM is a heterogeneous disease; emerging evidence has delineated distinct subtypes exhibiting varied clinical trajectories and differential responses to treatment [6]. The pathogenesis of T2DM primarily involves insulin resistance and/or impaired pancreatic β-cell function [7]. Current hypoglycemic agents include biguanides, sulfonylureas, glinides, and α-glucosidase inhibitors [8]. While these medications effectively control blood glucose levels, long-term use is often accompanied by adverse effects in clinical practice [9]. For example, biguanides may induce gastrointestinal disturbances such as nausea, vomiting, and diarrhea [10], whereas sulfonylureas, including glimepiride and glibenclamide, can precipitate severe hypoglycemia or exacerbate pancreatic β-cell dysfunction [11]. Consequently, the identification of alternative or adjunctive therapeutic strategies for T2DM has become a pressing research priority. Natural-product-derived bioactive compounds offer potential advantages, including reduced side effects and multi-target regulatory effects; however, systematic standardization is required to ensure reproducibility and precise dosing [12,13,14]. In recent years, these compounds have attracted considerable attention for their capacity to ameliorate T2DM, which is often accompanied by changes in the gut microbiota (GM). In T2DM, GM dysbiosis is typically characterized by an increased Bacillota/Bacteroidota ratio, reduced abundance of short-chain fatty acid (SCFA)-producing bacteria (e.g., *Alistipes*, *Akkermansia*), and overgrowth of opportunistic pathogens such as *Anaeroplasma* [15]. These alterations have been linked to insulin resistance and hyperglycemia through multiple mechanisms, including impaired SCFAs production, disruptions in bile acid metabolism, and increased endotoxemia [16].

*Chaga mushroom* is an edible fungus that primarily grows on birch trees in northern regions between latitudes of 40° and 60° N. It contains a diverse array of bioactive constituents, including polysaccharides, phenolic compounds (e.g., hispidin, hispidin analogs, protocatechuic acid), triterpenoids (e.g., betulinic acid, betulin), and melanin. These compounds exhibit varying biological activities, such as immunomodulatory, anti-inflammatory, antioxidant, hypoglycemic, and hypolipidemic effects [17]. However, most prior studies have focused on *Chaga mushroom* polysaccharides, obtained from the precipitate after water extraction–ethanol precipitation, which have demonstrated notable hypoglycemic effects [18]. In contrast, the corresponding supernatant, the by-product of this process, referred to as the supernatant of water extraction–ethanol precipitation of *Chaga mushroom* (SWEPC), has received limited attention. This by-product fraction is enriched in low-molecular-weight compounds (e.g., phenols, coumarins, carboxylic acids) that may possess bioactivity, and investigating it could enhance the comprehensive utilization of *Chaga mushroom* [19,20,21]. The chemical composition of crude extracts may vary due to factors such as the sources of raw materials, harvest time, and extraction procedures, inherently limiting batch-to-batch consistency. In this study, we used a single batch of *Chaga mushroom* and prepared SWEPC under strictly controlled conditions. Preliminary chemical characterization of SWEPC was performed using UPLC-QTOF-MS/MS, identifying hundreds of compounds and clarifying their major categories. Nevertheless, these findings represent only preliminary chemical profiles, and future studies on standardized cultivation, extraction optimization, and quality control systems remain necessary. In this study, we aimed to investigate the potential anti-hyperglycemic effects of SWEPC and its association with changes in the GM. Given the presence of multiple bioactive components, including low-molecular-weight phenols, coumarins, and possibly residual oligosaccharides, we focused on assessing the holistic effects of SWEPC on hyperglycemia and GM, rather than isolating a single mechanism.

## 2. Materials and Methods

### 2.1. SWEPC Preparation

The dried fruiting bodies of *Chaga mushroom* used in this study were obtained from a single batch, purchased from Bethune Herbs Enterprise Inc., Richmond Hill, ON, Canada, in October 2023. The extraction and preparation procedure was conducted as follows: The fruiting bodies were finely ground and sieved through a 40-mesh screen. A total of 100 g (dry weight) of the powder was mixed with distilled water at a ratio of 1:50 (g/mL) in a 10 L flat-bottomed flask equipped with a reflux condenser. The mixture was extracted under reflux at 60 °C for 6 h with magnetic stirring. The resulting extract was filtered through double-layered filter paper and concentrated under reduced pressure at 60 °C using a rotary evaporator (Shanghai Airang Instrument Co., Ltd., Shanghai, China) to one-fifth of the original volume (1 L). Subsequently, 3 L of anhydrous ethanol (Sinopharm Group, Beijing, China) was gradually added with stirring, and the mixture was allowed to precipitate overnight at 4 °C. The suspension was centrifuged at 4500 rpm for 15 min at 4 °C, and the supernatant was collected. The supernatant was then further concentrated under reduced pressure until dry, re-dissolved in 100 mL of distilled water, and subjected to lyophilization for 48 h to obtain SWEPC powder (4.31 g, 4.31% yield). The chemical composition of SWEPC was characterized via UPLC-QTOF-MS/MS, and the powder was stored in a desiccator protected from light until use.

### 2.2. Animal Experiment

**Figure 1:**
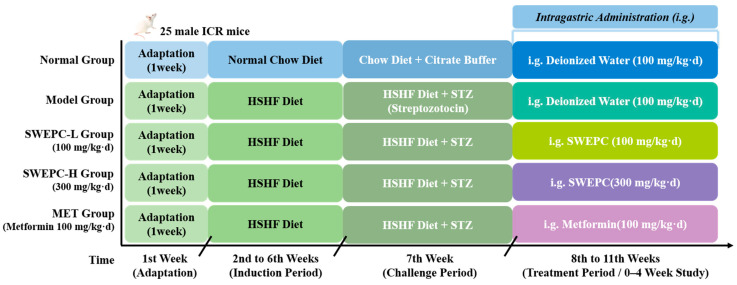
The experimental timeline for the animal study.

Figure 1 outlines the experimental timeline for the animal study. Twenty-five healthy male ICR mice (SPF grade, 4 weeks old, body weight [BW] 20 ± 3 g) were procured from the Medical College of Yangzhou University. The mice were housed in a climate-controlled environment (23 ± 2 °C, controlled humidity) under a 12-h light/dark cycle, with ad libitum access to food and water. Following a one-week acclimation period, five mice were randomly assigned to the Normal group and fed a standard diet. The remaining mice were provided a high-sugar and high-fat (HSHF) diet consisting of 58.8% standard feed, 15% sucrose, 15% lard, 1% cholesterol, 0.2% cholate, and 10% egg yolk; the diet composition percentages were based on weight/weight (*w*/*w*). The HSHF diet had an estimated caloric density of 4.5 kcal/g (fat ~35% of total energy), whereas the standard diet provided 3.85 kcal/g (fat ~10%). After four weeks on their respective diets, the HSHF-fed mice (*n* = 20) received intraperitoneal injections of streptozotocin (Sinopharm Group, Beijing, China) (45 mg/kg) on alternating days. Fasting blood glucose (FBG) levels were measured from tail vein blood 48 h after the third injection. Mice exhibiting FBG ≥ 11.1 mmol/L were considered successfully modeled for T2DM. These T2DM mice were randomly divided into four groups: Model (*n* = 5), SWEPC-L (100 mg/kg/day, *n* = 5), SWEPC-H (300 mg/kg/day, *n* = 5), and metformin hydrochloride (MET, 100 mg/kg/day, *n* = 5). Mice received their respective treatments via oral gavage daily for four weeks. Mice in the Normal and Model groups received an equivalent volume of deionized water. During the treatment period, the Model, SWEPC-L, SWEPC-H, and MET groups continued to receive the HSHF diet. Daily observations were conducted to monitor the mice’s fur condition, activity levels, and overall health, while daily food intake was recorded. BW measurements were performed following a 12-h fasting period at weeks 0, 2, and 4 during the intragastric administration period, with FBG levels assessed from tail-tip blood samples. At the conclusion of week 4, all mice underwent a 12-h fast prior to baseline blood glucose measurement (G_0h_). Subsequently, mice received a glucose solution (2 g/kg) via oral gavage for the oral glucose tolerance test (OGTT), with blood glucose levels recorded at 0.5 h (G_0.5h_), 1 h (G_1h_), and 2 h (G_2h_). The overall glucose excursion during OGTT was quantified by calculating the area under the curve (AUC) using the following formula:

AUC of OGTT = 0.25 × (G_0h_ + G_0.5h_) + 0.25 × (G_0.5h_ + G_1h_) + 0.5 × (G_1h_ + G_2h_).

Prior to dissection, mice underwent overnight fasting. Anesthesia was induced via the intraperitoneal administration of Sutai (Sinopharm Group, Beijing, China) (55 mg/kg) in combination with serazine hydrochloride (Sinopharm Group, Beijing, China) (5 mg/kg). Blood was then collected via eyeball extraction, followed by euthanasia through cervical dislocation. Liver, pancreatic, and cecal tissues were fixed in 4% paraformaldehyde, while remaining hepatic and cecal specimens were rapidly flash-frozen in liquid nitrogen and stored in an ultra-low-temperature freezer for subsequent analyses. All animal procedures adhered to the “Guidelines for the Care and Use of Laboratory Animals” published by the National Academy of Sciences of the National Institutes of Health (Publication No. 85-23, Revised Edition, 1985). Ethical approval for the experimental protocol was granted by the Laboratory Animal Ethics Committee of Jiangsu Medical Vocational College (Approval No.: SYLL-2024-711).

### 2.3. Serum Preparation

Blood samples were allowed to clot at room temperature for 2 h and subsequently centrifuged at 3000 rpm for 15 min at 4 °C. The resulting serum supernatant was collected and stored at −80 °C until analysis.

### 2.4. Homeostasis Model Assessment (HOMA) of Insulin-Related Indices

Fasting insulin (FINS) concentrations were determined using a commercial ELISA kit (Catalog No. CD21914, Purity Biotechnology Co., Ltd., Wuhan, China) according to the manufacturer’s instructions. In brief, 50 µL of standards or samples were added to wells coated with anti-insulin antibody and incubated at 37 °C for 30 min. After five washes with wash buffer, 50 µL of HRP-conjugated detection antibody was added and incubated for 30 min at 37 °C. Subsequently, 50 µL of chromogen solution was added and incubated in the dark at 37 °C for 15 min, followed by the addition of 50 µL of stop solution. Absorbance was measured at 450 nm within 15 min. Homeostatic model assessment (HOMA) indices were calculated based on FINS and FBG concentrations as follows:

HOMA-pancreatic islet β-cell function (HOMA-β) = 20 × FINs/(FBG-3.5); HOMA-insulin resistance (HOMA-IR) = FINs × FBG/22.5; and HOMA-insulin sensitivity (HOMA-IS) = 1/(FINs × FBG).

### 2.5. Biochemical Indicator Analysis in Serum and Liver

Approximately 100 mg of liver tissue was excised, rinsed with ice-cold 0.9% normal saline, blotted dry, and weighed. The tissue was homogenized in nine volumes (*w*/*v*) of ice-cold 0.9% normal saline using a motor-driven tissue homogenizer (JZQ-8, Shaoxing Supo Instrument Co., Ltd., Shaoxing, China) at 10,000 rpm for 30 s, repeated three times with 30-s intervals on ice. The homogenate was centrifuged at 4000 rpm for 15 min at 4 °C, and the supernatant was carefully collected and stored at −80 °C until further analysis. Serum and liver levels of low-density lipoprotein cholesterol (LDL-c), high-density lipoprotein cholesterol (HDL-c), glycated serum protein (GSP), total cholesterol (TC), and triglycerides (TG) were determined using commercial kits (Nanjing Jiancheng Institute of Bioengineering, Nanjing, China) according to the manufacturer’s instructions.

### 2.6. Analysis of qPCR

For RNA analysis, total RNA was extracted from 20 mg of liver tissue using 600 µL of TRIzol reagent (Invitrogen, Carlsbad, CA, USA) following the manufacturer’s protocol. cDNA synthesis was performed using the RevertAid First Strand cDNA Synthesis Kit (Takara, Kusatsu City, Japan). Quantitative PCR (qPCR) was conducted via SYBR^®^ Premix Ex Taq™ II (Takara, Japan) using an ABI 7500 fluorescence qPCR instrument (Applied Biosystems, Waltham, MA, USA), with five replicates per sample. All primers were synthesized by Sangon Biotech (Shanghai) Co., Ltd. (Shanghai, China), and the primer sequences are provided in Table 1. The qPCR program consisted of an initial denaturation at 95 °C for 5 min, followed by 40 cycles of 95 °C for 15 s and 60 °C for 60 s, concluding with a final extension at 60 °C for 5 min. Relative mRNA expression levels were calculated using the 2^−ΔΔCt^ method.

### 2.7. Microbiota Structure Analysis

Genomic DNA was extracted from intestinal contents using the cetyltrimethylammonium bromide (CTAB) method. The V3-V4 region of the 16S rRNA gene was amplified using primers 341F (5′-CCTAYGGGRBGCASCAG-3′) and 806R (5′-GGACTACNNGGGTATCTAAT-3′), synthesized by Sangon Biotech (Shanghai) Co., Ltd. Thermal cycling conditions included an initial denaturation at 98 °C for 1 min, followed by 30 cycles of 98 °C for 10 s, 50 °C for 30 s, and 72 °C for 30 s, with a final extension at 72 °C for 5 min. PCR products were mixed with an equal volume of 1× TAE buffer and visualized on a 2% agarose gel. Amplicons were combined in equimolar ratios and purified using the Universal DNA Purification Kit (DP214-02, TianGen Biotech, Beijing, China). Sequencing libraries were constructed using the NEB Next^®^ Ultra DNA Library Prep Kit (Illumina, San Diego, CA, USA) following the manufacturer’s protocol, with index codes added. Library quality was evaluated using an Agilent 5400 system (Agilent Technologies, Santa Clara, CA, USA), and sequencing was performed on an Illumina platform, generating 250 bp paired-end reads. Taxonomic annotation was performed via comparison with the Greengenes database.

### 2.8. Assessment of SCFAs in the Cecum

Acetic acid (AA), propionic acid (PA), and butyric acid (BA) standards were obtained from Anhui Zesun Technology Co., Ltd. (Hefei, China). A mixed standard solution at concentrations of 0.1, 0.5, 1, 5, 10, and 50 mmol/L was prepared to construct the calibration curve. For sample preparation, 200 mg of cecal contents was homogenized with deionized water, vortexed with 0.5 mL phosphoric acid for 2 min, and centrifuged at 4000 rpm for 20 min. The extraction was repeated by adding 1 mL of anhydrous ether to the supernatant. The supernatants were filtered through a 0.22 µm nylon membrane before analysis.

SCFAs were analyzed using a gas chromatograph (GC-2010Plus, Shimadzu, Kyoto, Japan) equipped with an HP-INNOWAX capillary column (30 m × 0.25 mm × 0.25 µm, Agilent Technologies, USA). The column flow rate was maintained at 1 mL/min using nitrogen as the carrier gas. The injection port was held at 260 °C, and 1 µL of sample was injected at a split ratio of 10:1. The oven temperature program was set as follows: an initial temperature of 100 °C for 1 min, ramped at 5 °C/min to 200 °C, and held for 2 min. Analytes were identified based on retention times, and quantification was performed using the external standard method.

### 2.9. Statistical Analysis

Each analysis was performed with a minimum of three technical replicates. Data are presented as mean ± standard deviation (SD) for *n* = 5 mice per group. Statistical significance was determined using one-way analysis of variance followed by Tukey’s post hoc test for multiple comparisons. When the assumptions of normality or homogeneity of variances were not met, the non-parametric Kruskal–Wallis test was applied. A *p*-value < 0.05 was considered statistically significant, with exact *p*-values reported in the results. All statistical analyses were conducted using SPSS 24.0 (IBM, New York, NY, USA) and GraphPad Prism 9.0 (GraphPad Software Inc., San Diego, CA, USA).

## 3. Results

### 3.1. Composition of SWEPC

The chemical composition of SWEPC was characterized using UPLC-QTOF-MS/MS (Table S1). A total of 495 compounds were identified, including 278 in positive ion mode (ESI^+^) and 217 in negative ion mode (ESI^−^). The predominant bioactive constituents comprised phenols (59 compounds), carboxylic acids and derivatives (30), coumarins and derivatives (48), and indoles and derivatives (53). Semi-quantitative analysis revealed that among coumarins, isofraxidin exhibited a peak area of 7.27 × 10^8^, scopoletin exhibited a peak area of 6.26 × 10^8^, esculetin exhibited a peak area of 1.73 × 10^7^, and fraxetin exhibited a peak area of 5.39 × 10^8^. Within the phenol/phenol ether class, eugenol (7.96 × 10^7^), coniferyl aldehyde (1.67 × 10^8^), and syringaldehyde (4.49 × 10^8^) were highly abundant. Notably, the carboxylic acid derivatives betaine (6.90 × 10^9^) and stachydrine (4.00 × 10^9^) were the most abundant ions detected. Additionally, indole derivatives such as tryptamine (4.97 × 10^7^) and 5-hydroxytryptophol (1.27 × 10^8^) were identified.

### 3.2. The Effects of SWEPC on Glycemic Parameters

**Figure 2:**
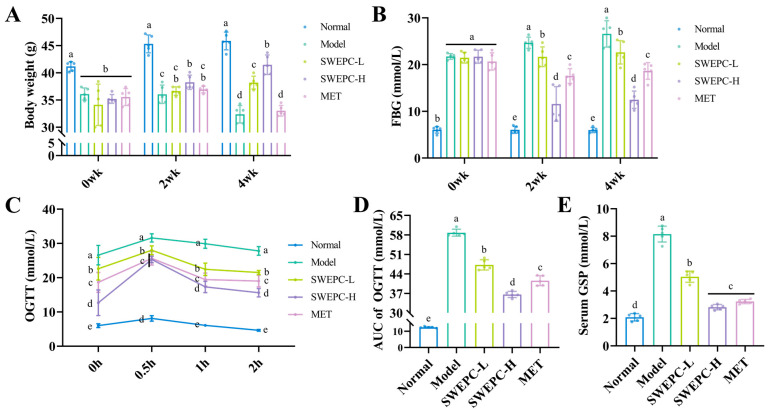
The effects of SWEPC treatment during the experimental period: (**A**) body weight, (**B**) FBG, (**C**) OGTT, (**D**) AUC of OGTT, and (**E**) serum GSP. SWEPC refers to the supernatant of water extraction–ethanol precipitation in *Chaga mushroom*, and T2DM indicates type 2 diabetes mellitus. WK stands for week. Data are presented as mean ± SD (*n* = 5 per group). Different superscript letters represent statistically significant differences between groups (*p* < 0.05).

As shown in Figure 2A, at week 0, BW declined markedly across all groups, except the Normal group. Following two weeks of SWEPC intervention, mice in the SWEPC-H group exhibited significantly higher BW compared with the Model group (*p* = 0.016). At week 4, BW in the SWEPC-H and SWEPC-L groups was substantially greater than that observed in the Model and MET groups, with the SWEPC-H group demonstrating significantly superior weight gain relative to the SWEPC-L group (*p* = 0.002).

Changes in FBG levels during SWEPC administration are presented in Figure 2B. At week 0, FBG levels in all groups, except the Normal group, were significantly elevated (*p* < 0.001). After two and four weeks of intervention, FBG levels in the SWEPC-treated groups were markedly lower than those in the Model group. Among them, the SWEPC-H group exhibited significantly reduced FBG compared with the SWEPC-L (*p* < 0.001) and MET (*p* = 0.004) groups, while the MET group displayed a significantly lower FBG than the SWEPC-L group (*p* = 0.041).

As depicted in Figure 2C, blood glucose peaked at 0.5 h following oral glucose administration and subsequently declined gradually. Glucose tolerance was quantitatively assessed by calculating the AUC of the OGTT (Figure 2D). Relative to the Model group, the AUC of OGTT decreased by 19.61%, 37.72%, and 29.27% in the SWEPC-L, SWEPC-H, and MET groups, respectively (*p* < 0.001). Serum GSP levels are shown in Figure 2E. The Model group exhibited significantly higher GSP levels compared with all other groups (*p* < 0.001). Furthermore, GSP levels in the SWEPC-H and MET groups were significantly lower than those in the SWEPC-L group (*p* < 0.001).

### 3.3. The Effects of SWEPC on Serum/Liver Indicators

**Figure 3:**
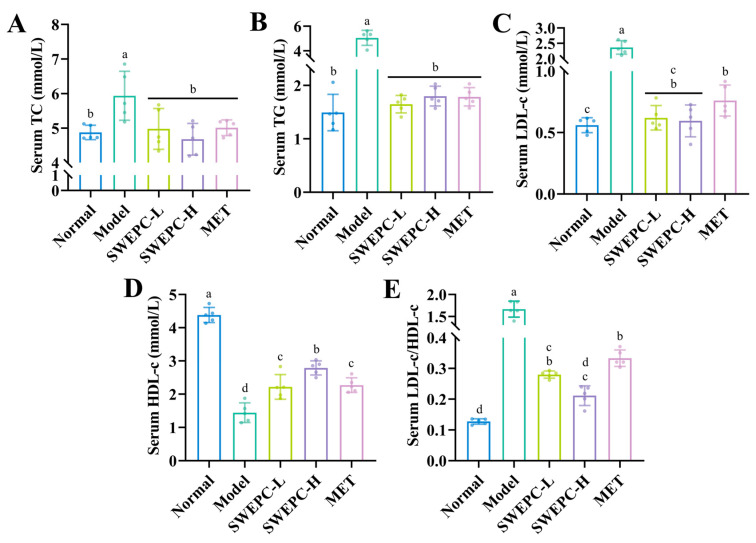
The effects of SWEPC intervention on serum biochemical indicators in mice. (**A**) TC, (**B**) TG, (**C**) LDL-c, (**D**) HDL-c, and (**E**) LDL-c/HDL-c sera. Data are presented as mean ± SD (*n* = 5 per group). Different superscript letters represent statistically significant differences between groups (*p* < 0.05).

Figure 3 illustrates the effects of SWEPC on serum lipid profiles. Compared with the Model group, the serum concentrations of TC, TG, LDL-c, and the LDL-c/HDL-c ratio were significantly reduced across all treated groups, whereas HDL-c levels were markedly increased (*p* < 0.001). Notably, serum HDL-c in the SWEPC-H group was significantly higher than those in the SWEPC-L (*p* = 0.003) and MET (*p* = 0.007) groups (Figure 3D), and the serum LDL-c/HDL-c ratio in the SWEPC-H group was significantly lower than that in the MET group (*p* = 0.033) (Figure 3E).

**Figure 4:**
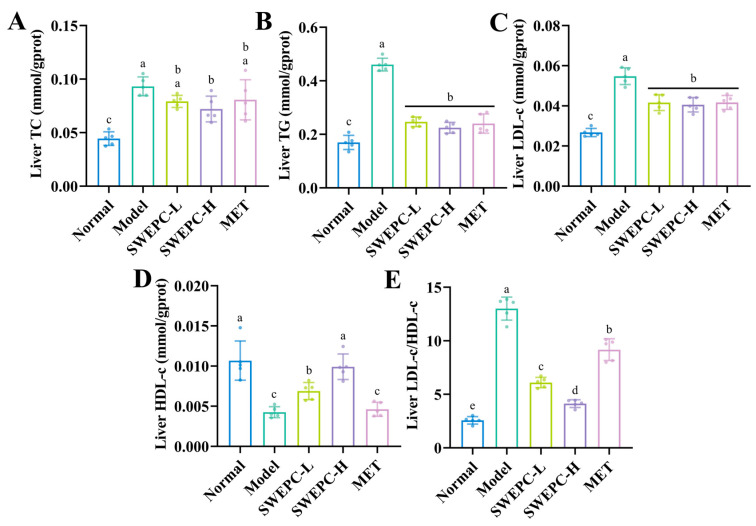
The effects of SWEPC intervention on liver biochemical indicators. The levels of TC (**A**), TG (**B**), LDL-c (**C**), HDL-c (**D**), and LDL-c/HDL-c (**E**) in the liver. Data are presented as mean ± SD (*n* = 5 per group). Different superscript letters represent statistically significant differences between groups (*p* < 0.05).

**Figure 5:**
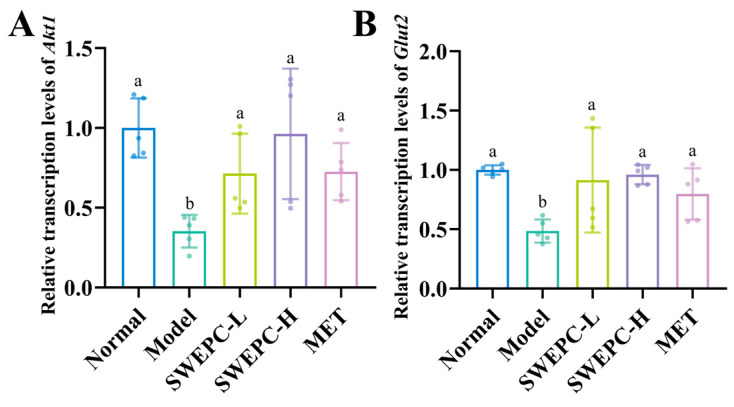
The effects of SWEPC on mRNA transcription in (**A**) *Akt1* and (**B**) *Glut2*. Data are presented as mean ± SD (*n* = 5 per group). Different superscript letters represent statistically significant differences between groups (*p* < 0.05).

As shown in Figure 4A, hepatic TC levels in the Normal (*p* < 0.001) and SWEPC-H (*p* = 0.008) groups were significantly lower than that in the Model group. Figure 4B,C,E demonstrated that hepatic TG, LDL-c, and LDL-c/HDL-c levels were markedly decreased in all treated groups relative to the Model group (*p* < 0.001). Importantly, the hepatic LDL-c/HDL-c ratio in the SWEPC-H group was significantly lower than those in the SWEPC-L and MET groups (*p* < 0.001), whereas the SWEPC-L group exhibited a lower ratio compared with the MET group (*p* < 0.001). As depicted in Figure 4D, hepatic HDL-c levels were significantly elevated in the SWEPC-L and SWEPC-H groups relative to the Model and MET groups, with the SWEPC-H group showing the greatest increase compared with the SWEPC-L group (*p* = 0.004). Figure 5A,B depict the effects of SWEPC on hepatic gene expression of Protein Kinase B (*Akt1*) and Glucose transporter-2 (*Glut2*). Compared with the Model group, the Normal, SWEPC-L, SWEPC-H, and MET groups exhibited significant upregulation of *Akt1* and *Glut2* mRNA levels.

### 3.4. The Influence of SWEPC on Pancreatic Islet Function Index

**Figure 6:**
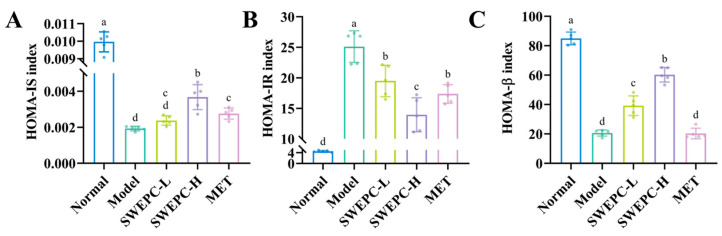
The effects of SWEPC intervention on pancreatic islet function. (**A**) HOMA-IS, (**B**) HOMA-IR, (**C**) HOMA-β. HOMA-IS: homeostatic model assessment–insulin sensitivity index; HOMA-IR: homeostatic model assessment–insulin resistance index; HOMA-β: homeostatic model assessment–pancreatic β-cell function index. Data are presented as mean ± SD (*n* = 5 per group). Different superscript letters represent statistically significant differences between groups (*p* < 0.05).

As shown in Figure 6A, the HOMA-IS index was significantly increased in the Normal, SWEPC-H, and MET groups relative to the Model group. Notably, HOMA-IS in the SWEPC-H group was significantly higher than those in the SWEPC-L (*p* < 0.001) and MET (*p* = 0.004) groups. Figure 6B shows that HOMA-IR indices were significantly reduced across all treated groups compared with the Model group, with the SWEPC-H group displaying a markedly lower HOMA-IR than the SWEPC-L (*p* = 0.001) and MET (*p* = 0.022) groups. As illustrated in Figure 6C, the HOMA-β index, reflecting pancreatic β-cell function, was significantly increased in the SWEPC-H and SWEPC-L groups compared with the Model and MET groups, with the SWEPC-H group exhibiting a significantly higher HOMA-β than the SWEPC-L group (*p* < 0.001).

### 3.5. Changes in GM and SCFAs in the Cecal Contents of Mice Following SWEPC Intervention

**Figure 7:**
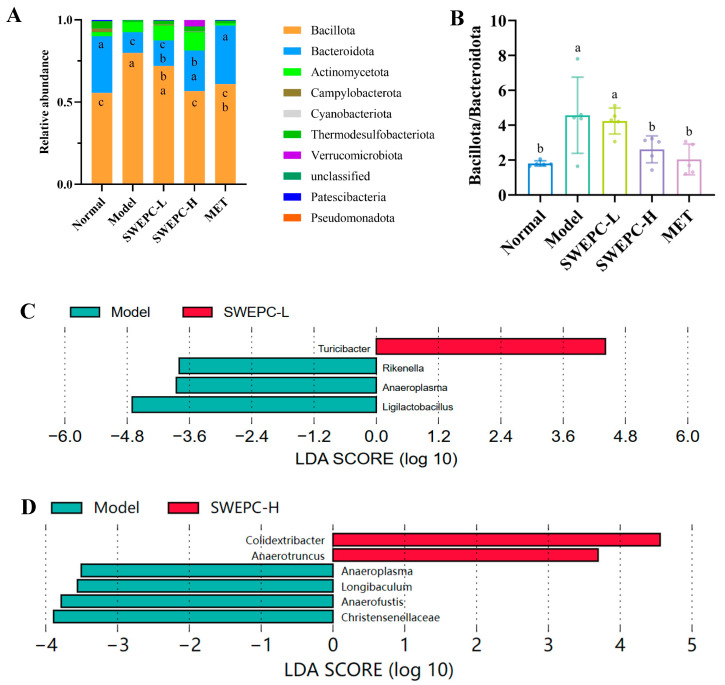
Changes in the cecal intestinal microbiota following SWEPC intervention. (**A**) The diversity of the gut microbiota at the phylum level. (**B**) The ratio of Bacillota to Bacteroidota. The expanded error bar chart shows significant differences in the horizontal intestinal microbiota between the two groups: (**C**) SWEPC-L and Model groups; (**D**) SWEPC-H and Model groups. Data are presented as mean ± SD (*n* = 5 per group). Different superscript letters represent statistically significant differences between groups (*p* < 0.05).

In this study, we assessed GM diversity at both the phylum and genus levels. At the phylum level, Bacillota and Bacteroidota constituted the two dominant bacterial communities, collectively exceeding 80% relative abundance (Figure 7A). Compared with the Model group, Bacillota abundance was significantly reduced in the Normal (*p* < 0.001), SWEPC-H (*p* = 0.001), and MET (*p* = 0.004) groups, whereas Bacteroidota abundance was markedly increased. As shown in Figure 7B, the Bacillota/Bacteroidota ratio was significantly decreased in the Normal (*p* = 0.001), SWEPC-H (*p* = 0.015), and MET (*p* = 0.002) groups relative to the Model group, with further reductions observed in the SWEPC-H (*p* = 0.038) and MET (*p* = 0.007) groups compared with the SWEPC-L group. At the genus level, differential bacterial abundances are illustrated in Figure 7C,D. Compared with the Model group, *Turicibacter* abundance was markedly increased in the SWEPC-L group, whereas *Ligilactobacillus*, *Anaeroplasma*, and *Rikenella* levels were significantly decreased (Figure 7C). In the SWEPC-H group (Figure 7D), *Anaerotruncus* and *Colidextribacter* levels were substantially increased, whereas *Christensenellaceae*, *Anaerofustis*, *Longibaculum*, and *Anaeroplasma* levels were significantly reduced relative to the Model group.

**Figure 8:**
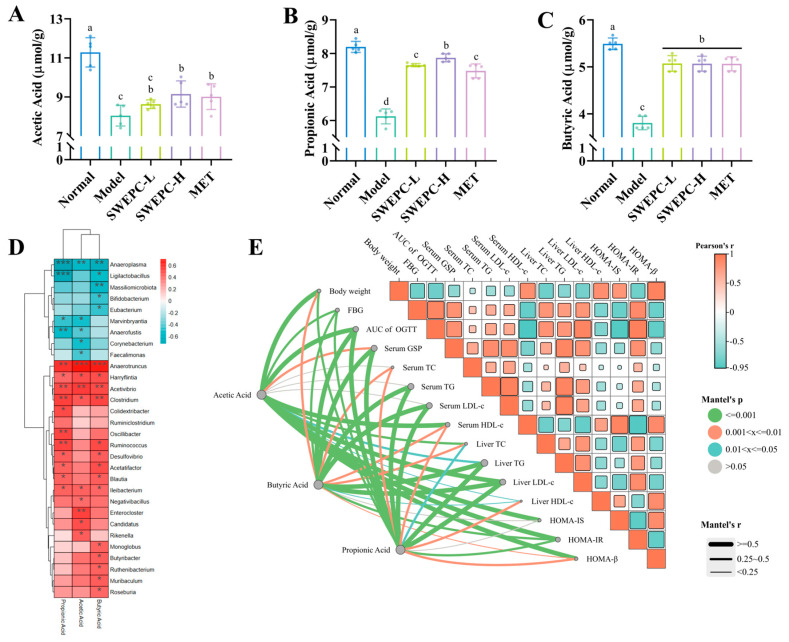
Changes in short-chain fatty acids in the cecal contents of mice following SWEPC intervention: (**A**) acetic acid; (**B**) propionic acid; (**C**) butyric acid. (**D**) Spearman correlation heatmap between bacterial genera and short-chain fatty acids. Red indicates a positive correlation, and teal indicates a negative correlation. (**E**) Mantel test results. Pearson correlation analysis was used to evaluate the relationship between blood glucose parameters and specific intestinal metabolites. Data are presented as mean ± SD (*n* = 5 per group). Different superscript letters represent statistically significant differences between groups (*p* < 0.05).* indicates *p* < 0.05, ** indicates *p* < 0.01, and *** indicates *p* < 0.001.

Figure 8A–C depict SCFAs concentrations across the groups. AA levels were significantly elevated in the Normal (*p* < 0.001), SWEPC-H (*p* = 0.008), and MET (*p* = 0.018) groups relative to the Model group (Figure 8A). PA concentrations were markedly increased in all treated groups (*p* < 0.001), with the SWEPC-H group exhibiting significantly higher PA levels than the SWEPC-L (*p* = 0.048) and MET (*p* = 0.001) groups (Figure 8B). BA levels were significantly increased in the Normal, SWEPC-L, SWEPC-H, and MET groups compared with the Model group (*p* < 0.001) (Figure 8C). Figure 8D illustrates correlations between GM genera and SCFAs levels. Positive associations with AA were observed for *Anaerotruncus*, *Harryflintia*, *Acetivibrio*, *Clostridium*, *Ilebacterium*, *Negativibacillus*, *Enterocloster*, *Candidatus*, and *Rikenella*, whereas *Anaeroplasma*, *Marvinbryantia*, *Anaerofustis*, *Corynebacterium*, and *Faecalimonas* were negatively correlated. Similarly, PA levels were positively associated with *Anaerotruncus*, *Harryflintia*, *Acetivibrio*, *Clostridium*, *Colidextribacter*, *Oscillibacter*, *Ruminococcus*, *Desulfovibrio*, *Acetifactor*, *Blautia*, and *Ilebacterium*, while PA levels negatively correlated with *Anaeroplasma*, *Ligilactobacillus*, *Marvinbryantia*, and *Anaerofustis*. For BA levels, positive correlations were observed with *Anaerotruncus*, *Harryflintia*, *Acetivibrio*, *Clostridium*, *Ruminococcus*, *Desulfovibrio*, *Acetifactor*, *Blautia*, *Ilebacterium*, *Monoglobus*, *Buturibacter*, *Ruthenibacterium*, *Muribaculum*, and *Roseburia*, whereas *Anaeroplasma*, *Ligilactobacillus*, *Massiliomicrobiota*, *Bifidobacterium*, and *Eubacterium* exhibited negative associations. Notably, *Anaerotruncus*, *Harryflintia*, *Acetivibrio*, *Clostridium*, and *Ilebacterium* were positively correlated with all three SCFAs (AA, PA, BA), while *Anaeroplasma* exhibited negative correlations with all three. Finally, the Mantel test was performed to evaluate correlations between blood glucose-related parameters and SCFAs levels (Figure 8E). Significant associations were observed between hypoglycemic parameters, including BW, FBG, AUC of OGTT, serum GSP, serum HDL-c, liver TC, TG, LDL-c, HDL-c, HOMA-IR, HOMA-β, and levels of AA, PA, and BA.

**Figure 9:**
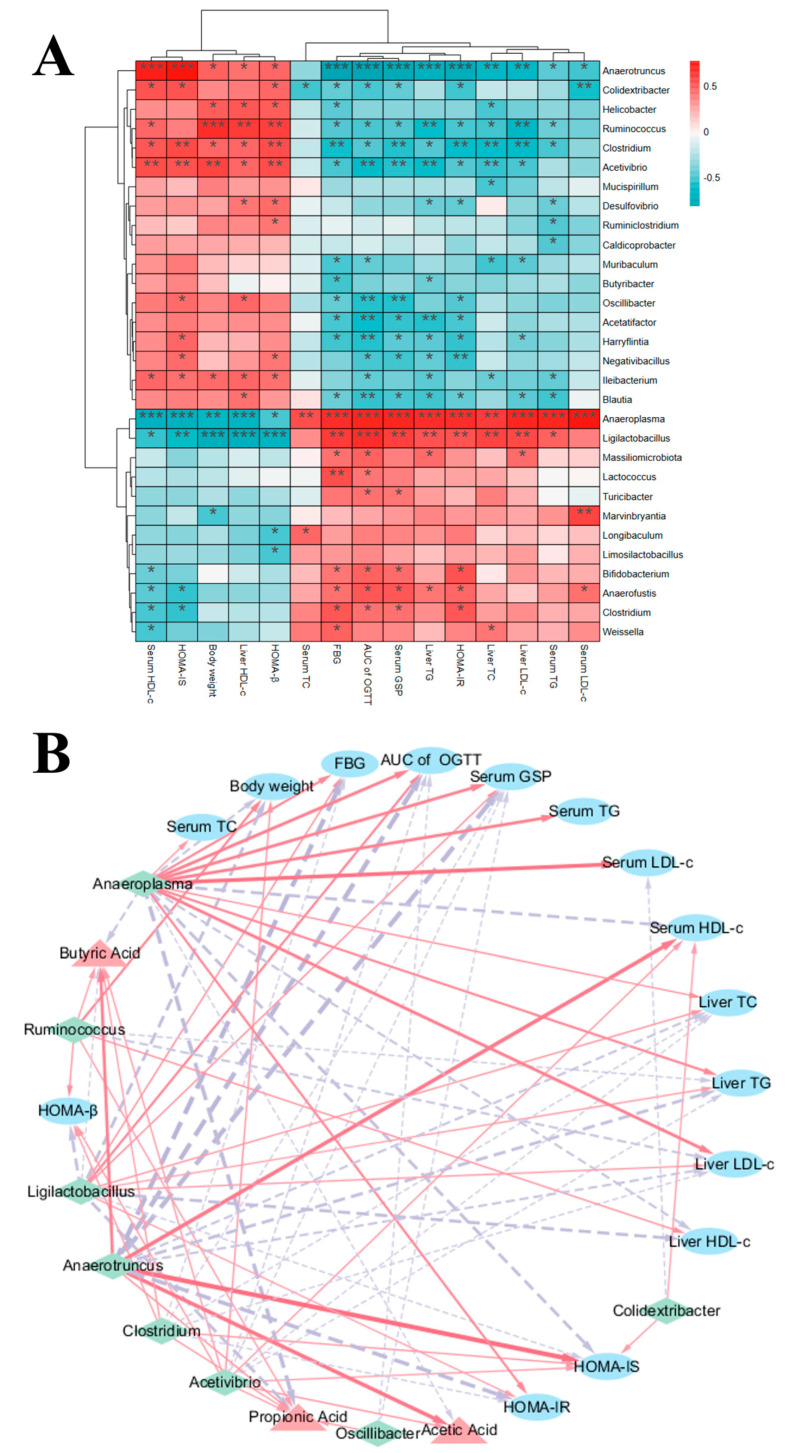
A hierarchical clustering analysis (**A**) and the visualization of the correlation network of the Spearman correlation analysis between hypoglycemic parameters and representative bacterial genera. In (**A**), red and teal, respectively, represent positive and negative correlations, and the depth of the color indicates the strength of the correlation. In (**B**), blue, green, and pastel pink, respectively, represent biochemical indicators, bacterial genera, and short-chain fatty acids. The red solid line indicates r > 0.5; the purple dotted line indicates r < −0.5. * indicates *p* < 0.05, ** indicates *p* < 0.01, and *** indicates *p* < 0.001.

Figure 9A illustrates the links between biochemical markers and bacterial genera. *Anaeroplasma* exhibited a positive relationship with serum TC, FBG, AUC of OGTT, serum GSP, liver TG, HOMA-IR, liver TC, liver LDL-c, serum TG, and serum LDL-c, whereas it displayed a negative relationship with serum HDL-c, HOMA-IS, BW, liver HDL-c, and HOMA-β. *Ligilactobacillus* demonstrated a positive relationship with FBG, AUC of OGTT, serum GSP, liver TG, HOMA-IR, liver TC, liver LDL-c, and serum TG and a negative relationship with serum HDL-c, HOMA-IS, BW, liver HDL-c, and HOMA-β. *Anaerotruncus* exhibited a positive relationship with serum HDL-c, HOMA-IS, BW, liver HDL-c, and HOMA-β and a negative relationship with FBG, AUC of OGTT, serum GSP, liver TG, HOMA-IR, liver TC, liver LDL-c, serum TG, and serum LDL-c. *Ruminococcus* displayed a positive relationship with serum HDL-c, BW, liver HDL-c, and HOMA-β and a negative relationship with FBG, AUC of OGTT, serum GSP, liver TG, HOMA-IR, liver TC, liver LDL-c, and serum TG. Figure 9B presents parameters with correlation coefficients of |r| > 0.5. *Anaerotruncus* was correlated with HOMA-IS (r = 0.772), serum HDL-c (r = 0.707), AA (r = 0.711), serum GSP (r = −0.758), AUC of OGTT (r = −0.788), HOMA-IR (r = −0.724), and FBG (r = −0.810), whereas *Anaeroplasma* was correlated with serum LDL-c (r = 0.748) and PA (r = −0.723).

## 4. Discussion

T2DM represents a chronic metabolic disorder primarily characterized by insulin resistance and impaired insulin secretion. Managing T2DM has emerged as a significant public health challenge in contemporary society [22]. Recent studies highlight the promising potential of natural bioactive compounds for the treatment of T2DM and the alleviation of insulin resistance. In this study, the effects of SWEPC on hyperglycemic manifestations in T2DM mice were evaluated. After four weeks, SWEPC treatment significantly increased BW and reduced FBG compared with the Model group. These results are consistent with the findings of Jiang et al., who reported improvements in BW and FBG levels in rats that received the petroleum ether extract of *Schisandra sphenanthera* [23]. These observations suggest that SWEPC may exert a potential hypoglycemic effect in T2DM mice, with the SWEPC-H group demonstrating substantially greater improvements in BW and FBG levels than the SWEPC-L group. The OGTT is a method widely employed for evaluating glucose tolerance that measures blood glucose levels at 0, 0.5, 1.0, and 2.0 h following glucose administration [24]. In this study, blood glucose peaked 0.5 h after glucose intake. At the 2-h time point, blood glucose levels in both the SWEPC-H and SWEPC-L groups were markedly lower than those in the Model group, indicating that SWEPC enhances glucose tolerance in T2DM mice. These findings are in agreement with Liu’s study, which demonstrated improvements in OGTT outcomes in T2DM mice treated with chitosan guanidine [25].

While FBG and OGTT provide snapshots of blood glucose at specific time points, they do not capture long-term glucose fluctuations. Serum GSP levels, in contrast, reflect glucose variability over the preceding 2–3 weeks and serve as a critical indicator of glucose homeostasis [26]. Following four weeks of SWEPC administration, serum GSP concentrations in both treatment groups were significantly reduced compared with that in the Model group, with the SWEPC-H group exhibiting a substantially greater decrease than the SWEPC-L group. These results are consistent with the findings of Li et al., who reported improvements in GSP levels in T2DM mice treated with pressed degreased walnut meal extracts [27]. Collectively, these observations suggest that SWEPC exerts a pronounced hypoglycemic effect in T2DM mice, with the SWEPC-H formulation demonstrating superior efficacy. According to Tang et al., TC, TG, LDL-c, and HDL-c are closely associated with the dysregulated hepatic metabolism observed in T2DM [28]. In this study, the serum TC, TG, LDL-c, and LDL-c/HDL-c ratios in the SWEPC-L and SWEPC-H groups were significantly decreased relative those in to the Model group, whereas HDL-c levels were markedly increased. Moreover, SWEPC treatment improved hepatic lipid profiles, with significant improvements observed in the TC, TG, LDL-c, HDL-c, and LDL-c/HDL-c ratios. Notably, the SWEPC-H group displayed markedly superior improvements in HDL-c and LDL-c/HDL-c levels compared with the SWEPC-L group, consistent with Lv et al.’s study on the effects of Tirzepatide on TG, TC, FBG, and HDL-c in patients with T2DM [29]. These findings indicate that SWEPC mitigates hepatic lipid metabolism disturbances in T2DM mice.

GLUT2, located in the plasma membrane, is essential for glucose uptake into cells [30]. The phosphoinositide 3-kinase/AKT1 pathway plays a central role in insulin signaling. Activation of this pathway typically suppresses hepatic gluconeogenesis and improves insulin sensitivity, while also regulating lipid metabolism through downstream effectors such as sterol regulatory element-binding protein 1c [31]. In this study, SWEPC administration significantly upregulated *Akt1* and *Glut2* mRNA transcription in the hepatic tissue of T2DM mice. Additionally, lipid metabolism disorders can further aggravate insulin resistance and impair insulin secretion [32]. The HOMA indices are commonly employed to evaluate pancreatic islet function. Here, insulin sensitivity, insulin resistance, and pancreatic β-cell function were assessed using the HOMA-IS, HOMA-IR, and HOMA-β indices. After four weeks of SWEPC treatment, the SWEPC-H group exhibited substantially better outcomes across the HOMA-IS, HOMA-IR, and HOMA-β indices relative to the SWEPC-L group. These results indicate that SWEPC-H more effectively enhances islet function in T2DM mice compared with SWEPC-L, aligning with the observations of Yin et al., in which silymarin improved pancreatic islet function in T2DM mice [33].

The GM exerts a profound influence on health and disease, mediating complex nutritional interactions with the host [34]. In this study, SWEPC-H significantly reduced the Bacillota/Bacteroidota ratio at the phylum level in T2DM mice. Yang et al. also reported that a high-fat diet was associated with a higher Bacillota/Bacteroidota ratio and systemic inflammation. Moreover, elevated Bacillota and decreased Bacteroides levels were correlated with insulin resistance [35]. At the genus level, SWEPC treatment was associated with a marked decrease in the relative abundance of *Anaeroplasma*, and with increased relative abundances of *Anaerotruncus* and *Turicibacter*. These findings are consistent with those of Liu et al., who found that treatment with medicinal and food homologous plants was associated with both improved GM composition and reduced *Anaeroplasma* abundance in T2DM mice [36]. Similarly, Wu et al. found that ethanol extracts of *Sargassum fusiforme* were associated with increased *Anaerotruncus* abundance in T2DM mice [37], and Xie et al. observed a similar association between metformin treatment and enhanced *Turicibacter* abundance in this model [38]. Notably, the functional roles of gut bacterial genera are context-dependent; our interpretations are based on correlations observed within these specific T2DM mice.

Indigestible dietary carbohydrates, including dietary fiber, resistant starch, and oligosaccharides, are fermented by anaerobic bacteria to produce SCFAs, primarily AA, BA, and PA [39]. SCFAs have been shown to improve insulin sensitivity by stimulating insulin secretion and promoting pancreatic β-cell cluster expansion [40]. In this study, SWEPC-treated mice had higher intestinal levels of AA, BA, and PA compared with the Model group, and *Anaerotruncus* abundance was positively correlated with these SCFAs, whereas *Anaeroplasma* showed a negative correlation. Yao et al. reported that *Cyclocarya paliurus* polysaccharide was associated with increased levels of AA and BA in the intestine, and that *Anaerotruncus* abundance was correlated with these changes. These SCFAs have also been associated with beneficial effects on obesity, T2DM, allergies, and irritable bowel syndrome [41]. Although our study did not directly test such a causal relationship, the observed positive correlation between *Anaerotruncus* abundance and SCFAs aligns with those findings. To further explore the relationship between blood glucose-related parameters and GM composition, hierarchical clustering analysis was performed. *Anaeroplasma* showed strong positive correlations with serum TC, FBG, OGTT AUC, serum GSP, liver TG, HOMA-IR, liver TC, liver LDL-c, serum TG, and serum LDL-c, as well as strong negative correlations with serum HDL-c, HOMA-IS, BW, liver HDL-c, and HOMA-β. Zhou et al. reported that *Anaeroplasma* was associated with inflammatory responses [42]. In contrast, *Anaerotruncus* exhibited strong positive associations with serum HDL-c, HOMA-IS, BW, liver HDL-c, and HOMA-β and negative associations with FBG, OGTT AUC, serum GSP, liver TG, HOMA-IR, liver TC, liver LDL-c, serum TG, and serum LDL-c; Yang et al. noted that *Anaerotruncus* was associated with anti-inflammatory effects [43]. *Ruminococcus* was positively correlated with serum HDL-c, BW, liver HDL-c, and HOMA-β and negatively correlated with FBG, OGTT AUC, serum GSP, liver TG, HOMA-IR, liver TC, liver LDL-c, and serum TG. Deng et al. reported that *Ruminococcus* was associated with FBG and inflammation [44], while Dai et al. found that increased *Ruminococcus* abundance in T2DM rats treated with mulberry leaf polysaccharides was correlated with alleviation of hyperglycemic symptoms [45]. Furthermore, the strong correlations between the relative abundances of *Anaerotruncus* and *Anaeroplasma* and blood glucose-related parameters suggest that these bacterial genera are associated with T2DM-related metabolic traits. In addition, chemical profiling of SWEPC revealed the presence of multiple bioactive compounds previously associated with antidiabetic or GM-modulating activities. Among these, isofraxidin [46] and scopoletin [47] are known to inhibit α-glucosidase and improve insulin resistance, whereas esculetin [48] and fraxetin [49] exhibit antioxidant and anti-inflammatory effects. Highly abundant phenolic compounds, including eugenol, coniferyl aldehyde, and syringaldehyde, have been reported to possess hypoglycemic and anti-inflammatory properties [50]. Notably, betaine, the most abundant compound detected, has been associated with improved insulin sensitivity and increased relative abundance of *Akkermansia* [51]. Additionally, indole derivatives have been suggested to be associated with gut barrier function via aryl hydrocarbon receptor signaling [52]. Collectively, these findings suggest that SWEPC contains multiple bioactive constituents that may synergistically contribute to the observed improvements in hyperglycemia and alterations in GM composition, although the specific contributions of each compound warrant further investigation.

Notably, the associations observed between GM composition and blood glucose parameters are correlative and do not establish causality. This study represents an initial exploration of SWEPC’s role in modulating GM in T2DM mice. To establish causative relationships, future investigations should employ fecal microbiota transplantation, antibiotic-mediated microbial depletion, and comprehensive validation of microbial metabolites beyond SCFAs. Furthermore, the mechanistic analysis in the current study is preliminary, as only two genes (*Akt1* and *Glut2*) were examined. Future research should incorporate more comprehensive molecular approaches, such as transcriptomics or proteomics, to better understand the underlying mechanisms. It is also noteworthy that SWEPC contains phenolic compounds with anti-disaccharidase and anti-lipase activities; thus, changes in GM may partially result from increased fermentable substrates reaching the colon rather than a direct prebiotic effect. Measuring residual cecal nutrients (fat, carbohydrate, protein) is recommended in future studies. Additional limitations and future directions include the following: (i) batch-to-batch variability in SWEPC was minimized by using a single batch with chemical profiling, but future research should focus on isolating active components and establishing quality control standards; (ii) the HSHF diet may dilute protein and micronutrients, potentially confounding observed effects, necessitating the use of fortified control diets; (iii) SWEPC may contain soluble minerals leached from the *Chaga mushroom*, which could contribute to the observed effects, highlighting the need for mineral profiling and mineral-matched controls; (iv) the fatty acid composition and endogenous cholesterol content of lard and egg yolk in the HSHF diet were not characterized, potentially increasing total dietary cholesterol beyond 1%; (v) gut tissue-specific markers of microbiota–host interaction should be examined; and (vi) lack of control groups (HSHF diet + SWEPC, and normal diet + SWEPC) to distinguish SWEPC’s antidiabetic actions from general metabolic effects of the diet or in healthy mice.

## 5. Conclusions

In this study, we investigated the changes in GM composition in T2DM mice following SWEPC intervention and examined the associations between SWEPC administration and T2DM-related metabolic improvements. The results showed that SWEPC-H treatment was associated with a marked reduction in the Bacillota/Bacteroidota ratio at the phylum level in T2DM mice. At the genus level, SWEPC-H treatment was associated with the relative abundances of *Anaerotruncus*, *Turicibacter*, and *Anaeroplasma*. These microbial shifts were observed alongside changes in hyperglycemic symptoms in T2DM mice. Furthermore, SWEPC treatment significantly improved varied metabolic parameters, including BW, FBG, AUC of OGTT, serum GSP, TC, TG, LDL-c, HDL-c, and HOMA indices (HOMA-IR, HOMA-IS, and HOMA-β). In the cecal contents, SWEPC increased the levels of AA, PA, and BA, with PA exhibiting the most pronounced increase in the SWEPC-H group. Chemical profiling revealed that SWEPC contains bioactive compounds, including isofraxidin, scopoletin, and betaine, which have previously been associated with antidiabetic and GM-modulating activities. Notably, *Anaeroplasma*, *Anaerotruncus*, and *Ruminococcus* displayed correlations with multiple blood glucose-related parameters, suggesting that these genera may be of interest for future mechanistic studies on T2DM pathogenesis and disease progression. These findings suggest that SWEPC treatment is associated with mitigation of hyperglycemic symptoms in T2DM mice, and this effect is closely associated with changes in GM composition. In this study, we provide preliminary experimental evidence supporting the potential role of SWEPC in alleviating hyperglycemia in T2DM mice; however, further validation in larger cohorts, over longer durations, and using standardized extracts is required before translational applications can be considered.

## Figures and Tables

**Table 1:** A list of all primers used for qPCR.

Primer Name	Forward Primer (5′-3′)	Reverse Primer (5′-3′)
*β-* *a* *c* *t* *in*	TGTCCACCTTCCAGCAGATGT	AGCTCATAACAGTCCGCCTAGA
*A* *kt* *1*	ACTCATTCCAGACCCACGAC	CCGGTACACCACGTTCTTCT
*G* *lut* *2*	TACGGCAATGGCTTTATC	CCTCCTGCAACTTCTCAAT

## Data Availability

The original contributions presented in this study are included in the article/Supplementary Material. Further inquiries can be directed to the corresponding authors.

## Supplementary Materials

The following supporting information can be downloaded at: https://www.mdpi.com/article/10.3390/foods15132383/s1, Table S1: The chemical composition of SWEPC.

## Abbreviations

The following abbreviations are used in this manuscript:

T2DM	type 2 diabetes mellitus
FBG	fasting blood glucose
GM	gut microbiota
SCFAs	short-chain fatty acids
AA	acetic acid
BA	butyric acid
PA	propionic acid
SWEPC	the supernatant of water extraction–ethanol precipitation of *Chaga mushroom*
BW	body weight
MET	metformin hydrochloride
OGTT	oral glucose tolerance test
AUC	area under the curve
FINS	fasting insulin
HOMA-β	HOMA-pancreatic islet β-cell function
HOMA-IR	HOMA-insulin resistance
HOMA-IS	HOMA-insulin sensitivity
LDL-c	low-density lipoprotein cholesterol
HDL-c	high-density lipoprotein cholesterol
GSP	glycated serum protein
TC	total cholesterol
TG	triglycerides
*A**kt**1*	protein kinase B
*G**lut**2*	glucose transporter-2
HOMA	homeostasis model assessment

## References

[1] Chaudhry M., Sif S. (2026). Epigenetic regulation in type II diabetes: Linking molecular mechanisms to clinical management. J. Diabetes Metab. Disord..

[2] Yao Y., Wu X., Wu H., Su W., Li P. (2026). Multi-omics analyses unveil the effects of a long-term high-salt, high-fat, and high-fructose diet on rats. Foods.

[3] Duncan B.B., Magliano D.J., Boyko E.J. (2026). IDF diabetes atlas 11th edition 2025: Global prevalence and projections for 2050. Nephrol. Dial. Transpl..

[4] Eisner D.C., Day J. (2026). Type 5 diabetes mellitus: An atypical variant returns. JNP-J. Nurse Pract..

[5] Fei Z., Xu Y., Zhang G.Y., Liu Y., Li H., Chen L.X. (2024). Natural products with potential hypoglycemic activity in T2DM: 2019–2023. Phytochemistry.

[6] Weber K.S., Schlesinger S., Lang A., Straßburger K., Maalmi H., Zhu A., Zaharia O.P., Strom A., Bönhof G.J., Goletzke J. (2024). Association of dietary patterns with diabetes-related comorbidities varies among diabetes endotypes. Nutr. Metab. Cardiovas..

[7] Ilgaz B., Akoglu G., Alioglu A., Soyocak A., Ates F.S.O., Argun D. (2025). Investigation of the association between MSR1 serum levels and type 2 diabetes: A preliminary study. Ir. J. Med. Sci..

[8] Laiteerapong N., Alexander J., Philipson L., Winn A.N., Huang E.S. (2023). First-line therapy for type 2 diabetes with sodium–glucose cotransporter-2 inhibitors and glucagon-like peptide-1 receptor agonists. Ann. Intern. Med..

[9] Prasathkumar M., Becky R., Anisha S., Dhrisya C., Sadhasivam S. (2022). Evaluation of hypoglycemic therapeutics and nutritional supplementation for type 2 diabetes mellitus management: An insight on molecular approaches. Biotechnol. Lett..

[10] Al Zoubi M.S., Al Kreasha R., Aqel S., Saeed A., Al-Qudimat A.R., Al-Zoubi R.M. (2024). Vitamin B12 deficiency in diabetic patients treated with metformin: A narrative review. Ir. J. Med. Sci..

[11] Alwafi H., Wong I.C.K., Naser A.Y., Banerjee A., Mongkhon P., Whittlesea C., Alsharif A., Wei L. (2024). Concurrent use of oral anticoagulants and sulfonylureas in individuals with type 2 diabetes and risk of hypoglycemia: A UK population-based cohort study. Front. Med..

[12] Tudu M., Samanta A. (2023). Natural polysaccharides: Chemical properties and application in pharmaceutical formulations. Eur. Polym. J..

[13] Xue H.K., Hao Z.T., Gao Y.C., Cai X., Tang J.T., Liao X.J., Tan J.Q. (2023). Research progress on the hypoglycemic activity and mechanisms of natural polysaccharides. Int. J. Biot. Macromol..

[14] Fan Y.Q., Liu Y.Q., Wu Y., Dai F.F., Yuan M.Q., Wang F.Y., Bai Y., Deng H.B. (2021). Natural polysaccharides based self-assembled nanoparticles for biomedical applications—A review. Int. J. Biol. Macromol..

[15] Ye X.W., Wu K.F., Xu L.Y., Cen Y.X., Ni J.H., Chen J.Y., Zheng W.X., Liu W. (2023). Methanol extract of *Inonotus obliquus* improves type 2 diabetes mellitus through modifying intestinal flora. Front. Endocrinol..

[16] Shalbaf N., Sadeghi S., Homaee S., Saberian F. (2025). Probiotics, prebiotics, synbiotics, and FMT for glycemic control: A systematic review of clinical efficacy and mechanistic readouts in type 2 diabetes and related dysglycemia. Metab. Open.

[17] Wang Y.M., Gu J.S., Wu J.Y., Xu Y.X., Liu Y.T., Li F.X., Liu Q., Lu K.L., Liang T., Hao J.W. (2025). Natural products and health care functions of *Inonotus obliquus*. Curr. Issues Mol. Biol..

[18] Lu Y.P., Jia Y.N., Xue Z.H., Li N.N., Liu J.Y., Chen H.X. (2021). Recent developments in *Inonotus obliquus* (*Chaga mushroom*) polysaccharides: Isolation, structural characteristics, biological activities and application. Polymers.

[19] Shahidi F., Ambigaipalan P. (2015). Phenolics and polyphenolics in foods, beverages and spices: Antioxidant activity and health effects. J. Funct. Foods.

[20] Venugopala K.N., Rashmi V., Odhav B. (2013). Review on natural coumarin lead compounds and their pharmacological activity. Biomed. Res. Int..

[21] Godlewska-Żyłkiewicz B., Świsłocka R., Kalinowska M., Golonko A., Świderski G., Arciszewska Ż., Nalewajko-Sieliwoniuk E., Naumowicz M., Lewandowski W. (2020). Biologically active compounds of plants: Structure-related antioxidant, microbiological and cytotoxic activity of selected carboxylic acids. Materials.

[22] Roesti E.S., Boyle C.N., Zeman D.T., Sande-Melon M., Storni F., Cabral-Miranda G., Knuth A., Lutz T.A., Vogel M., Bachmann M.F. (2020). Vaccination against amyloidogenic aggregates in pancreatic islets prevents development of type 2 diabetes mellitus. Vaccines.

[23] Jiang H.H., Feng S.B., Zhang P.P., Wang J.J., Jiang Y., Zhang H.W., Song X.M., Huang W.L., Xie Y.D., Deng C. (2024). Petroleum ether extract of *Schisandra sphenanthera* prevents hyperglycemia and insulin resistance in association with modulation of sweet taste receptors and gut microbiota in T2DM rats. J. Ethnopharmacol..

[24] Hua K.F., Zhang M.Y., Zhang Y., Ren B.J., Wu Y.H. (2022). Characteristics of OGTT and correlation between the insulin to c-peptide molar ratio, HOMA-IR, and insulin antibodies in T2DM patients. Diabetes Metab. Syndr. Obes..

[25] Liu Y.C., Miao Q.Y., Liu Y., Jiang M.M. (2024). Effects of chitosan guanidine on blood glucose regulation and gut microbiota in T2DM. Int. J. Biol. Macromol..

[26] Wu J.P., Xie L.D., Qu Z.Y., Song H., Sun X.M., Hu Y., Li W.L. (2025). Exploring the active components of hypoglycemic effect in different polar fractions of *Inonotus obliquus* based on spectrum-effect relationship. J. Funct. Foods.

[27] Li Y.L., Chen D., Zhang F., Lin Y.P., Ma Y.G., Zhao S.L., Chen C.Y., Wang X.S., Liu J. (2020). Preventive effect of pressed degreased walnut meal extracts on T2DM rats by regulating glucolipid metabolism and modulating gut bacteria flora. J. Funct. Foods.

[28] Tang J., Lin Z.W., Liu X.D., Li B., Wu X.L., Lv J., Qi X., Lin S., Dai C.Q., Li T. (2024). Analyzing the changing trend of corneal biomechanical properties under different influencing factors in T2DM patients. Sci. Rep..

[29] Lv X.Y., Wang H., Chen C.Y., Zhao Y.T., Li K., Wang Y.W., Wang L.T., Fu S.B., Liu J.F. (2024). The effect of tirzepatide on weight, lipid metabolism and blood pressure in overweight/obese patients with type 2 diabetes mellitus: A systematic review and meta-analysis. Diabetes Metab. Syndr. Obes..

[30] He X.Y., Wang C.E., Zhu Y.X., Jiang X.Q., Qiu Y.Y., Yin F., Xiong W.Y., Liu B., Huang Y. (2022). Spirulina compounds show hypoglycemic activity and intestinal flora regulation in type 2 diabetes mellitus mice. Algal Res..

[31] Zhang Z., Liang X., Tong L.J., Lv Y.Y., Yi H.X., Gong P.M., Tian X.Y., Cui Q.Y., Liu T.J., Zhang L.W. (2021). Effect of *Inonotus obliquus* (Fr.) Pilat extract on the regulation of glycolipid metabolism via PI3K/Akt and AMPK/ACC pathways in mice. J. Ethnopharmacol..

[32] Wang K.X., Cui Y.X., Lin P., Yao Z.N., Sun Y. (2021). JunD regulates pancreatic β-Cells function by altering lipid accumulation. Front. Endcrinol..

[33] Yin S., Zhu F.Y., Liu Y., Chen Q. (2025). Effects of silymarin on insulin resistance and sensitivity: A systematic review and meta-analysis of randomized controlled trials. Diabetes Res. Clin. Pract..

[34] Zou Y.F., Song X.J., Liu N., Sun W., Liu B. (2022). Intestinal flora: A potential new regulator of cardiovascular disease. Aging Dis..

[35] Yang X.M., Gu Y.P., Liu H.Z., Shang F.F., Koyama T. (2024). Modulation of the gut microbiota alleviates insulin resistance in type 2 diabetic mice by daucosterol from eleocharis dulcis peel. J. Funct. Foods.

[36] Liu W.T., Zhang Y.K., Zheng M.Z., Ye Y.X., Shi M.J., Wang X., Cao L.Y., Wang L. (2024). Polysaccharides in medicinal and food homologous plants regulate intestinal flora to improve type 2 diabetes: Systematic review. Phytomedicine.

[37] Wu S.Y., Zuo J.H., Cheng Y., Zhang Y., Zhang Z.S., Wu M.J., Yang Y., Tong H.B. (2021). Ethanol extract of *Sargarsum fusiforme* alleviates HFD/STZ-induced hyperglycemia in association with modulation of gut microbiota and intestinal metabolites in type 2 diabetic mice. Food Res. Int..

[38] Xie Y.H., Li X.X., Meng Q.S., Li J.J., Wang X., Zhu L.Y., Wang W.W., Li X.Q. (2024). Interplay between gut microbiota and tryptophan metabolism in type 2 diabetic mice treated with metformin. Microbiol. Spectr..

[39] He M., Wei W.Q., Zhang Y.C., Xiang Z.X., Peng D., Kasimumali A., Rong S. (2024). Gut microbial metabolites SCFAs and chronic kidney disease. J. Transl. Med..

[40] Münte E., Hartmann P. (2025). The role of short-chain fatty acids in metabolic dysfunction-associated steatotic liver disease and other metabolic diseases. Biomolecules.

[41] Yao Y., Yan L.J., Chen H., Wu N., Wang W.B., Wang D.S. (2020). *Cyclocarya paliurus* polysaccharides alleviate type 2 diabetic symptoms by modulating gut microbiota and short-chain fatty acids. Phytomedicine.

[42] Zhou R.R., He D., Zhang H.C., Xie J., Zhang S.H., Tian X.F., Zeng H.L., Qin Y.H., Huang L.Q. (2023). Ginsenoside Rb1 protects against diabetes-associated metabolic disorders in kkay mice by reshaping gut microbiota and fecal metabolic profiles. J. Ethnopharmacol..

[43] Yang X., Xin Y.J., Gu Y.Z., Wang Y.L., Hu X.J., Ying G.H., Zhang Q., He X.L. (2024). Total alkaloids of aconitum carmichaelii debx alleviate cisplatin-induced acute renal injury by inhibiting inflammation and oxidative stress related to gut microbiota metabolism. Phytomedicine.

[44] Deng J., Zou X.Y., Liang Y.X., Zhong J., Zhou K., Zhang J.W., Zhang M., Wang Z.Y., Sun Y.M., Li M.Y. (2023). Hypoglycemic effects of different molecular weight konjac glucomannans via intestinal microbiota and SCFAs mediated mechanism. Int. J. Biol. Macromol..

[45] Dai H.Y., Shan Z.Y., Shi L., Duan Y.H., An Y.C., He C.H., Lyu Y., Zhao Y.G., Wang M.L., Du Y.H. (2024). *Mulberry leaf* polysaccharides ameliorate glucose and lipid metabolism disorders via the gut microbiota-bile acids metabolic pathway. Int. J. Biol. Macromol..

[46] Li J., Li X.F., Li Z.K., Zhang L., Liu Y.G., Ding H., Yin S.Y. (2017). Isofraxidin, a coumarin component improves high-fat diet-induced hepatic lipid homeostasis disorder and macrophage inflammation in mice. Food Funct..

[47] Jang J.H., Park J.E., Han J.S. (2018). Scopoletin inhibits α-glucosidase in vitro and alleviates postprandial hyperglycemia in mice with diabetes. Eur. J. Pharmacol..

[48] Prabakaran D., Ashokkumar N. (2012). Antihyperglycemic effect of esculetin modulated carbohydrate metabolic enzymes activities in streptozotocin induced diabetic rats. J. Funct. Foods.

[49] Murali R., Srinivasan S., Ashokkumar N. (2013). Antihyperglycemic effect of fraxetin on hepatic key enzymes of carbohydrate metabolism in streptozotocin-induced diabetic rats. Biochimie.

[50] Jiang Y.G., Feng C.X., Shi Y.H., Kou X.R., Le G.W. (2022). Eugenol improves high-fat diet/streptomycin-induced type 2 diabetes mellitus (T2DM) mice muscle dysfunction by alleviating inflammation and increasing muscle glucose uptake. Front. Nutr..

[51] Sun Y.L., Li S.P., Liu R.X., Ma N., Zhao X.H., He X. (2025). A novel daidzein-betaine cocrystal alleviates obesity by improving lipid metabolism, modulating inflammation, and reshaping gut microbiota. Food Biosci..

[52] Wang A.R., He D.S., Wang T.Q., Guan C., Mu G.Q., Tuo Y.F. (2025). *Lactiplantibacillus plantarum* DPUL-S164 regulates aryl hydrocarbon receptor signaling to ameliorate dextran sodium sulfate-induced intestinal barrier damage by producing indole-3-lactic acid in a tryptophan-rich diet. Food Sci. Hum. Wellness.

