# Supplementary material for: Supernatant of Water Extraction–Ethanol Precipitation of Chaga mushroom Improves Hyperglycemia in Type 2 Diabetes Mellitus and Is Accompanied by Changes in Gut Microbiota Composition
Source: Foods. 2026 Jul 3;15(13):2383. doi: 10.3390/foods15132383 (PMC13361204; doi:10.3390/foods15132383)
Supplement: Supplementary file 1 [file foods-15-02383-s001.zip › Table S1.pdf]

Met ID	m/z	Retention Tim	Metabolite Name	Class	A2
M141T358_pos	141.0661687	358.279	(1-Methyl-1H-imidazol-4-yl)acetic acid	Azoles	29608715.46
M265T378_pos	265.0082533	378.162	(6-Thien-2-ylimidazo[2,1-b][1,3]thiazol-3-yl)acetic acid	Azoles	6174871.377
M97T264_pos	97.07650307	264.418	1,2-Dimethylimidazole	Azoles	45984545.45
M113T146_pos	113.0712313	146.056	1-(2-Hydroxyethyl)pyrazole	Azoles	12148938.93
M251T321_pos	251.0185913	321.267	1-(3-Bromophenyl)-3,5-dimethylpyrazole	Azoles	116088792.2
M281T521_pos	280.9903544	521.294	1-(4-Bromophenyl)-5-methyl-1H-pyrazole-4-carboxylic acid	Azoles	6922137.219
M244T278_pos	244.0499011	278.066	1-(4-Fluorophenyl)-5-methyl-1H-1,2,3-triazole-4-carboxylic acid	Azoles	5820918.213
M281T120_pos	281.101929	120.431	1-(4-Nitrophenyl)-3-phenyl-1H-pyrazol-5-ylamine	Azoles	16882518.64
M111T332_pos	111.0919721	332.313	1-(Propan-2-yl)-1H-imidazole	Azoles	5205836.706
M139T264_2_pos	139.1230537	264.418	1-Butyl-3-methyl-1H-imidazol-3-ium cation	Azoles	16815368.21
M125T170_pos	125.1075527	170.1515	1-Butylimidazole	Azoles	15083490.04
M111T120_pos	111.0556892	119.613	1-acetylimidazole	Azoles	46415819.84
M154T421_pos	154.1339438	421.3325	1-tert-Butyl-3-methyl-1H-pyrazol-5-ylamine	Azoles	18884800.93
M135T499_pos	135.0666234	499.2	1H,1'H-2,2'-Biimidazole	Azoles	2527420.861
M225T35_pos	225.0429529	35.2743	1H-Pyrazole-3-carboxylic acid, 5-(2,4-difluorophenyl)-	Azoles	29629489.22
M174T277_1_pos	174.1027255	277.283	2-(1H-Pyrazol-1-yl)benzylamine	Azoles	2575059.633
M141T330_pos	141.102261	330.338	2-(2-Methyl-1H-imidazol-1-yl)-1-propanol	Azoles	7917814.632
M259T496_pos	259.0213997	496.11	2-(3-Chlorophenyl)-4-methyl-1H-imidazole-5-carboxylic acid	Azoles	5100876.009
M155T318_pos	155.1179108	318.087	2-(Trimethyl-1H-pyrazol-4-yl)ethan-1-ol	Azoles	11045292.38
M139T960_pos	139.1230745	959.946	3,5-Dimethyl-4-propyl-1H-pyrazole	Azoles	57593728.27
M174T229_pos	174.0762317	229.247	3-Methyl-1-(4-nitrophenyl)-1H-pyrazol-5-ol	Azoles	12924339.07
M141T346_2_pos	141.1022852	346.355	3-tert-Butyl-5-isoxazolylamine	Azoles	8571581.831
M127T372_pos	127.0503822	371.784	4-Imidazoleacetic acid	Azoles	18758028.08
M139T345_pos	139.0502727	345.376	4-Imidazoleacrylic acid	Azoles	38839722.45
M83T433_pos	83.06092081	433.3	4-Methyl-1H-pyrazole	Azoles	4627386.808
M151T116_pos	151.0614695	116.089	5-(2-Furyl)-4H-1,2,4-triazol-3-amine	Azoles	88099358.66
M267T438_pos	267.0144541	438.449	5-Amino-4-cyano-1-(2,4-dichlorophenyl)-3-methyl-1H-pyrazole	Azoles	3482309.495
M151T144_pos	151.0866822	144.482	5-Butyl-1H-pyrazole-3-carboxylic acid	Azoles	18950423.36
M142T159_pos	142.0612809	159.036	Dimetridazole	Azoles	4078283.444
M239T31_1_pos	239.0338589	31.4836	Methyl 2-amino-5-(3-fluorophenyl)-1,3-thiazole-4-carboxylate	Azoles	31724316.58
M379T414_pos	379.0640687	414.363	Methyl 5-(4-methylsulfanylphenyl)-1-(4-trifluoromethyl-phenyl)-1H-pyrazole-3-carboxylate	Azoles	10815759.62
M361T392_pos	361.0553483	391.768	Pyraclofos	Azoles	14641556.9
M235T235_pos	235.1190147	235.187	Zolpidem	Azoles	37985421.37
M197T87_pos	197.0445554	87.45825	1-(5-Chloro-1H-benzimidazol-2-yl)ethanol	Benzimidazoles	13595299.57
M409T276_pos	409.3422573	276.364	1-[1-(1-Methylcyclooctyl)-4-piperidinyl]-2-[(3R)-3-piperidinyl]-1H-benzimidazole	Benzimidazoles	2388667.988
M231T309_pos	231.1816139	308.789	2-Octyl-1H-benzimidazole	Benzimidazoles	905337.3563
M229T352_pos	229.0343337	352.448	5,6-Dichloro-1-ethyl-2-methyl-1H-benzo[d]imidazole	Benzimidazoles	16703763.52
M224T375_pos	224.0917619	375.3285	Methyl 4-(5-nitro-1H-benzimidazol-2-yl)phenyl ether	Benzimidazoles	7840254.134
M256T381_pos	256.1179688	381.083	N-[1-(1H-Benzimidazol-2-yl)-3-methyl-1H-pyrazol-5-yl]acetamide	Benzimidazoles	85651233.63
M307T26_pos	307.0449176	25.8769	[(1-Phenyl-1H-benzimidazol-2-yl)sulfanyl]acetic acid	Benzimidazoles	21621319.52
M179T220_pos	179.0702537	219.808	3-Ketocarbofuran	Benzofurans	102938861.4
M196T332_pos	196.0728646	331.696	4-(1-Benzofuran-2-yl)pyridine	Benzofurans	24919515.73
M271T447_pos	270.9614646	446.522	5-Bromo-7-methoxy-1-benzofuran-2-carboxylic acid	Benzofurans	12735188.98
M353T398_1_pos	353.0855416	398.438	Griseofulvin	Benzofurans	38358524.43
M215T372_2_pos	215.1027784	372.1985	1H-2-Benzopyran-1-one, 3,4-dihydro-8-hydroxy-3-(4-hydroxypentyl)-	Benzopyrans	12060929.38
M175T276_pos	175.0714589	276.364	3,6-Dimethylchromone	Benzopyrans	12323367.56
M247T391_pos	247.0600193	390.9655	3,7,8-Trihydroxy-3-methyl-1,4-dihydropyrano[4,3-b]chromen-10-one	Benzopyrans	25584457.41
M309T31_pos	309.060418	31.4836	3,7,8-Trihydroxy-3-methyl-10-oxo-1,4-dihydropyrano[4,3-b]chromene-9-carboxylic acid	Benzopyrans	74403949.71
M225T166_pos	225.0757374	166.435	4,8-Dihydroxy-6-methoxy-3-methyl-3,4-dihydroisochromen-1-one	Benzopyrans	44698561.49
M221T92_pos	221.1172545	92.3728	6-Acetyl-5.8-dimethoxy-2.2-dimethyl-2H-1-benzopyran	Benzopyrans	24412605.6
M191T302_pos	191.0339149	301.552	Chromone-3-carboxylic acid	Benzopyrans	15092793.77
M235T103_pos	235.0601401	102.6459	Cimifugin	Benzopyrans	50516416.06
M491T435_pos	491.0581944	435.062	Cromolyn	Benzopyrans	6366191.086
M261T376_pos	261.0756781	376.306	Khellin	Benzopyrans	36825973.34
M245T402_2_pos	245.1496231	401.964	Koninginin D	Benzopyrans	14806263.29
M161T250_pos	161.0596714	249.689	Mellein	Benzopyrans	92864660.31
M269T413_pos	269.0422062	413.442	Sydowinin A	Benzopyrans	22892588.24
M118T338_2_pos	118.0864438	338.0475	Betaine	Carboxylic_acids_and_derivatives	6903792751
M144T337_pos	144.101931	336.8675	Stachydrine	Carboxylic_acids_and_derivatives	4002225860
M143T402_pos	143.0816061	402.337	Ectoine	Carboxylic_acids_and_derivatives	1604376519
M229T366_pos	229.1546537	365.901	Pro-Leu	Carboxylic_acids_and_derivatives	1274699728
M132T335_pos	132.1019964	335.234	N-Acetyl-DL-leucine	Carboxylic_acids_and_derivatives	1146214466
M344T95_pos	344.2279035	95.18445	N-(1-Amino-3,3-dimethyl-1-oxobutan-2-yl)-1-pentyl-1H-indole-3-carboxamide	Carboxylic_acids_and_derivatives	806463144.7
M406T35_1_pos	406.2528428	34.98695	Echinulin	Carboxylic_acids_and_derivatives	624765872.5
M116T376_3_pos	116.0708498	376.3625	DL-arginine	Carboxylic_acids_and_derivatives	457003629.7
M118T56_pos	118.0865481	55.7799	DL-Norvaline	Carboxylic_acids_and_derivatives	408566870.1
M160T363_pos	160.0968859	362.541	Betonicine	Carboxylic_acids_and_derivatives	271376530.9
M154T97_pos	154.04997	97.327	3-Succinimidopropionic acid	Carboxylic_acids_and_derivatives	267192530.2
M170T77_pos	170.1176294	76.6923	Trandolaprilat	Carboxylic_acids_and_derivatives	262993777
M100T63_pos	100.0761351	62.8982	5-Aminopentanamide	Carboxylic_acids_and_derivatives	180245846.8
M163T384_pos	163.0752138	384.348	.alpha.-Methylcinnamic acid	Cinnamic_acids_and_derivatives	28704702.61
M131T322_pos	131.0492404	321.528	1-O-Cinnamoylglucose	Cinnamic_acids_and_derivatives	5970967.443
M152T420_pos	152.0707323	419.833	2-Propenamide, N-(2,3-dihydro-1,4-benzodioxin-6-yl)-3-[4-(1,1-dimethylethyl)phenyl]-, (2E)	Cinnamic_acids_and_derivatives	12154024.58
M561T275_pos	561.1581216	274.753	Picroside III	Cinnamic_acids_and_derivatives	37583005.33
M361T254_1_pos	361.0916115	254.368	Rosmarinic acid	Cinnamic_acids_and_derivatives	76451618.05
M179T273_pos	179.0703227	273.151	Sinapine cation	Cinnamic_acids_and_derivatives	65343834.94
M329T262_pos	329.101809	261.9	Specioside	Cinnamic_acids_and_derivatives	48456431.97
M491T310_pos	491.1544626	309.945	[(2R,3S,4S,5R,6R)-3,4,5-Trihydroxy-6-[2-(3-hydroxy-5-oxooxolan-3-yl)propoxy]oxan-2-yl]methyl (E)-3-(4-hydroxyphenyl)prop-2-enoate	Cinnamic_acids_and_derivatives	14839799.4
M149T252_pos	149.0597358	251.687	trans-cinnamate	Cinnamic_acids_and_derivatives	97618613.3
M449T415_pos	449.1175582	415.25	4-(3,4-Dihydroxyphenyl)-7-hydroxy-2-oxo-2H-chromen-5-yl .beta.-D-glucopyranoside	Coumarins_and_derivatives	8389955.459
M387T481_pos	387.0874342	481.455	5,8-Dihydroxy-6-methoxy-2-oxo-2H-chromen-7-yl .beta.-D-glucopyranoside	Coumarins_and_derivatives	1303601.749
M207T255_1_pos	207.0651975	255.038	6,7-Dimethylesculetin	Coumarins_and_derivatives	454807769.8
M407T309_pos	407.0969773	309.167	6,7-dimethoxy-8-[(2s,3r,4s,5s,6r)-3,4,5-trihydroxy-6-(hydroxymethyl)oxan-2-yl]oxychromen-2-one	Coumarins_and_derivatives	62989912.91
M507T264_pos	507.1493596	264.198	6-Methoxy-2-oxo-2H-chromen-7-yl 3-O-acetyl-6-O-(6-deoxy-.alpha.-L-mannopyranosyl)-.beta.-D-glucopyranoside	Coumarins_and_derivatives	16176811.57
M219T268_pos	219.0652584	268.299	8-Acetyl-7-hydroxy-4-methylcoumarin	Coumarins_and_derivatives	44183108.59
M219T84_pos	219.065262	84.49015	8-Acetyl-7-methoxycoumarin	Coumarins_and_derivatives	181354356.3
M221T27_pos	221.0446099	26.758	8-Methoxycoumarin-3-carboxylic acid	Coumarins_and_derivatives	36163800.86
M313T412_pos	313.0683946	411.623	Aflatoxin B1	Coumarins_and_derivatives	25384050.87
M331T28_2_pos	331.0812263	28.3312	Aflatoxin G2	Coumarins_and_derivatives	21551670.54
M315T27_2_pos	315.0861327	26.7929	Aflatoxin b2	Coumarins_and_derivatives	24132305.24
M219T256_2_pos	219.0651445	255.643	Butanoic acid, 3-methyl-, 2-hydroxy-1-[hydroxy(7-methoxy-2-oxo-2H-1-benzopyran-6-yl)methyl]-2-methylpropyl ester	Coumarins_and_derivatives	44608243.84
M137T290_pos	137.05981	289.815	Coumafuryl	Coumarins_and_derivatives	68078528.87
M179T85_pos	179.033954	85.1899	Esculetin	Coumarins_and_derivatives	17268400.47
M149T35_1_pos	149.0234437	34.65885	Fraxetin	Coumarins_and_derivatives	538830054.2
M223T208_pos	223.0599555	208.375	Fraxidin	Coumarins_and_derivatives	38845947.03
M249T100_pos	249.0757159	100.201	Methyl 7-methoxycoumarin-4-acetate	Coumarins_and_derivatives	138999969.7
M245T386_pos	245.1131392	385.704	Osthole	Coumarins_and_derivatives	11564596.93
M457T302_pos	457.1749339	302.494	Trioxsalen	Coumarins_and_derivatives	2329483.954
M309T498_pos	309.116757	498.187	Warfarin	Coumarins_and_derivatives	1395466.246
M455T500_pos	455.1158108	500.072	[3-hydroxy-1-(4-methoxy-7-oxofuro[3,2-g]chromen-9-yl)oxy-3-methylbutan-2-yl] (e)-2-methylbut-2-enoate	Coumarins_and_derivatives	1256889.583
M192T266_pos	192.0655401	266.058	(2-oxo-2,3-dihydro-1h-indol-3-yl)acetic acid	Indoles_and_derivatives	9369415.753
M146T325_pos	146.0600449	324.918	(2E)-3-(1H-Indol-2-yl)-2-propenoic acid	Indoles_and_derivatives	12674596.13
M130T140_pos	130.0652974	140.112	2-(1H-Indol-1-yl)acetamide	Indoles_and_derivatives	12608459.83
M247T322_pos	247.0236907	322.078	3-Chloro-6-nitro-9H-carbazole	Indoles_and_derivatives	31046367.62
M146T72_pos	146.0601534	72.372	3-Hydroxy-3-methyl-2,3-dihydro-1H-indol-2-one	Indoles_and_derivatives	91774238.8
M443T505_pos	443.205222	504.651	3-[1-[3-(Dimethylamino)propyl]-5-methoxy-1H-indol-3-yl]-4-(1H-indol-3-yl)-1H-pyrrole-2,5-dione	Indoles_and_derivatives	17430530.84
M156T228_pos	156.0655982	227.8565	4,6-Difluoro-2,3-dihydro-1H-indole	Indoles_and_derivatives	23908217.08
M194T320_pos	194.0449032	320.407	5,6-Dihydroxyindole-2-carboxylic acid	Indoles_and_derivatives	23499280.55
M319T503_2_pos	319.0060275	503.284	5-Bromo-3-hydroxy-3-(2-pyridinylmethyl)-1,3-dihydro-2H-indol-2-one	Indoles_and_derivatives	6265564.987
M169T168_pos	169.0734505	168.111	5-carboline	Indoles_and_derivatives	22501387.2
M178T55_1_pos	178.0863286	54.70925	5-hydroxytryptophol	Indoles_and_derivatives	127331343.4
M219T402_pos	219.134052	402.317	6-Fluoro-3-(4-piperidinyl)-1H-indole	Indoles_and_derivatives	2839066.556
M230T383_pos	230.1135194	382.581	6-Methyl-2,3,4,9-tetrahydro-1H-carbazole-1-carboxylic acid	Indoles_and_derivatives	6859873.908
M205T324_pos	205.0972285	324.376	D-tryptophan	Indoles_and_derivatives	3586492.581
M188T324_pos	188.0706806	323.9215	DL-Indole-3-lactic acid	Indoles_and_derivatives	8250334.304
M232T478_pos	232.1656136	477.869	Dihydro-.beta.-erythroidine	Indoles_and_derivatives	36637985.63
M118T339_2_pos	118.0650953	339.292	Indole	Indoles_and_derivatives	29096364.88
M276T386_pos	276.1440781	385.976	Mahanimbine	Indoles_and_derivatives	27141491.5
M233T467_pos	233.12436	467.357	Melatonin	Indoles_and_derivatives	520119.7411
M354T195_pos	354.1905099	195.102	Methanone, [1-(4-hydroxypentyl)-1H-indol-3-yl](4-methyl-1-naphthalenyl)-	Indoles_and_derivatives	34017432.57
M169T271_pos	169.0655394	270.78	Methanone, [1-(5-fluoro-4-hydroxypentyl)-1H-indol-3-yl](4-methyl-1-naphthalenyl)-	Indoles_and_derivatives	23265480.52

M219T42_pos	219.1129482	42.4939	N-Acetyl-5-hydroxytryptamine	Indoles_and_derivatives	175864590.4
M357T403_pos	357.0814331	403.086	N-Phthalyl-L-tryptophan	Indoles_and_derivatives	14806983.89
M169T140_pos	169.0751316	140.112	Norharmane	Indoles_and_derivatives	44077727.05
M347T963_pos	347.2075508	963.411	Roxindole	Indoles_and_derivatives	10827006.34
M283T364_2_pos	283.0811361	363.669	Toceranib	Indoles_and_derivatives	34848678.43
M144T325_1_pos	144.0808205	324.918	Tryptamine	Indoles_and_derivatives	49719505.69
M352T440_pos	352.1268466	440.161	Verruculogen	Indoles_and_derivatives	7209234.807
M337T617_pos	337.1604216	616.682	Vincosamide	Indoles_and_derivatives	119396.1399
M237T291_pos	237.1232682	290.665	.beta.-Ala-Phe	Peptidomimetics	6871208.412
M308T37_2_pos	308.2945878	36.51485	13-(Dodecan-2-yl)-6-(1-hydroxyethyl)-3-(hydroxymethyl)-12-methyl-9-(propan-2-yl)-1-oxa-4,7,10-triazacyclotridecane-2,5,8,11-tetrone	Peptidomimetics	91564917.46
M226T164_pos	226.0685941	164.23	2-(4-Chloro-2-methylphenoxy)-N-(2-hydroxyethyl)acetamide	Phenol_ethers	6204447.127
M128T540_pos	128.1071846	540.435	4-[3-(4-Butoxyphenoxy)propyl]morpholine	Phenol_ethers	4986036.216
M115T40_1_pos	115.0545511	39.8599	5-[3-(Trifluoromethoxy)phenyl]furan-2-carbaldehyde	Phenol_ethers	281337423.7
M293T508_pos	293.0350929	507.93	Dimethyl 3-(3-chlorophenoxy)-2-oxopropylphosphonate	Phenol_ethers	4131381.109
M334T247_pos	334.310359	247.054	N,N-Dimethyl-N-(2-phenoxyethyl)-1-dodecanaminium cation	Phenol_ethers	59184855.97
M108T498_pos	108.055963	498.187	N-(4-Methoxybenzyl)-2-pyrimidinamine	Phenol_ethers	10959149.84
M324T310_pos	324.1665033	309.595	Pentamidine	Phenol_ethers	1344253.408
M246T722_pos	246.1812403	722.412	Tramadol	Phenol_ethers	704524.609
M181T317_pos	181.028502	317.227	[(4-Methoxyphenyl)thio]acetic acid	Phenol_ethers	74223365.21
M121T40_pos	121.0650345	40.27115	trans-Anethole	Phenol_ethers	142896342.5
M184T329_pos	184.096881	329.018	(-)-Epinephrine	Phenols	5340460.484
M235T28_1_pos	235.0238096	27.6075	2,4-Thiazolidinedione, 5-[[5-(4-fluoro-2-hydroxyphenyl)-2-furanyl]methylene]-, (5Z)-	Phenols	14248749.34
M140T301_pos	140.0468062	300.696	2,6-Dimethoxyphenol	Phenols	16090821.02
M153T132_pos	153.0560174	131.8355	2-Hydroxy-4-methoxybenzaldehyde	Phenols	129469971.1
M185T41_1_pos	185.080837	40.9183	3,4,5-Trimethoxyphenol	Phenols	66197211.98
M155T364_2_pos	155.0702939	363.669	3,4-Dimethoxyphenol	Phenols	24307223.91
M137T108_pos	137.0598427	107.849	3-Allyl-4-hydroxy-5-methoxybenzaldehyde	Phenols	31088485.08
M108T289_pos	108.0447046	289.148	3-Amino-4-fluorophenol	Phenols	2649387.563
M109T363_pos	109.0651463	362.9295	3-Methylphenol	Phenols	54518064.94
M126T95_pos	126.0552095	94.5602	4-Amino-1,2-benzenediol	Phenols	35206079.68
M137T131_pos	137.0598033	131.307	4-Hydroxy-3-methoxybenzenemethanol	Phenols	152462307.6
M197T40_pos	197.1173419	39.69365	5-(4-Hydroxypentyl)benzene-1,3-diol	Phenols	170869718.9
M140T107_pos	140.0707354	107.204	5-Amino-2-methoxyphenol	Phenols	86642646.74
M152T99_pos	152.0707239	99.4639	Acetaminophen	Phenols	16333593.36
M123T387_pos	123.0442595	387.198	Bilobol	Phenols	72502795.51
M131T271_2_pos	131.0492637	270.78	Coniferyl alcohol	Phenols	10731534.35
M179T251_pos	179.0702669	251.02	Coniferyl aldehyde	Phenols	166862562
M308T122_pos	308.2219489	122.327	Dihydrocapsaicin	Phenols	11500622.18
M182T61_pos	182.1177029	61.4459	Etilefrine	Phenols	8433377.453
M137T56_pos	137.0598131	56.42235	Eugenol	Phenols	79554103
M141T402_pos	141.0547129	401.537	Gentisyl alcohol	Phenols	58024837.68
M111T41_1_pos	111.044381	41.3633	Hydroquinone	Phenols	187567940.2
M194T252_pos	194.1176042	252.391	Isoproterenol	Phenols	105735364.1
M441T415_pos	441.0796411	415.25	Mirin	Phenols	14943904.28
M418T170_pos	418.3310179	169.517	N-Oleoyldopamine	Phenols	66787803.09
M184T39_3_pos	184.1697081	38.5041	Nonanamide, N-[(4-hydroxy-2-iodo-5-methoxyphenyl)methyl]-8-methyl-	Phenols	53865905.35
M95T40_pos	95.04961026	39.972	Phenol	Phenols	606222050.9
M313T270_pos	313.1067935	269.896	Puerol B	Phenols	44203073.07
M209T338_pos	209.080779	338.44	Sinapaldehyde	Phenols	23215879.24
M183T101_pos	183.0653413	101.485	Syringaldehyde	Phenols	448532462.6
M277T348_pos	277.1756968	347.858	[6]-Gingerol	Phenols	2367914.756
M174T379_pos	174.1237713	378.7115	1-(1-Benzylpiperidin-4-yl)-4-(methylsulfonyl)piperazine	Piperidines	123418189.9
M250T519_1_pos	250.1648744	518.58	1-Benzyl-4-phenyl-4-piperidinol	Piperidines	186755757.9
M146T143_pos	146.0812542	142.559	1-Deoxynojirimycin	Piperidines	16754573.89
M386T330_pos	386.1474079	329.718	1-{4-[(Diethylamino)sulfonyl]-2-nitrophenyl}-4-piperidinecarboxylic acid	Piperidines	2309624.526
M226T129_pos	226.1802104	128.549	2,2,6,6-Tetramethyl-4-piperidinyl 2-methylacrylate	Piperidines	83367346.03
M109T79_pos	109.0763755	79.36215	2,6-Piperidinedicarbonitrile	Piperidines	6547953.53
M100T37_pos	100.0761346	37.0778	3-Piperidone	Piperidines	10175316.64
M128T281_pos	128.0708035	281.4275	4-Methylpiperidine-2,6-dione	Piperidines	8218069.107
M129T328_pos	129.1023967	327.608	4-Piperidinecarboxamide	Piperidines	15095770.25
M158T280_pos	158.1176945	280.1885	Ethyl 3-piperidinecarboxylate	Piperidines	6172589.988
M173T309_pos	173.1286513	308.789	Ethyl 4-amino-1-piperidinecarboxylate	Piperidines	13222113.26
M173T211_pos	173.0923437	210.716	Glutethimide	Piperidines	23712131.63
M183T207_pos	183.1493065	206.54	N,N'-Bis(2,2,6,6-tetramethyl-4-piperidinyl)urea	Piperidines	32037848.99
M169T294_pos	169.1335561	294.209	N-(1-Adamantyl)-N'-(1-propyl-4-piperidinyl)urea	Piperidines	33327027.2
M143T313_pos	143.1179879	312.64	N-(4-Piperidinyl)acetamide	Piperidines	7931485.898
M116T785_pos	116.1072551	784.845	N-Methyl-N-(tetrahydro-2-furanylmethyl)-4-piperidinamine	Piperidines	6883749.603
M453T276_pos	453.2705309	276.364	Repaglinide	Piperidines	5642296.613
M293T307_pos	293.1857226	306.724	Thioperamide	Piperidines	25318050.53
M260T514_pos	260.1968291	514.406	Vesamicol	Piperidines	12040125.1
M256T317_pos	256.1291309	316.616	tert-Butyl 4-fluoro-4-(hydroxymethyl)piperidine-1-carboxylate	Piperidines	10314030.66
M265T391_pos	265.0705344	391.397	3-Hydroxy-2,9,10-trimethoxy-5,6-dihydroisoquinolino[3,2-a]isoquinolinium cation	Protoberberine_alkaloids_and_derivatives	25244647.84
M192T295_pos	192.1019397	294.894	Rotundine	Protoberberine_alkaloids_and_derivatives	21688651.32
M137T57_pos	137.0710791	57.152	3,4-Dimethyl-1H,6H-pyrano[2,3-c]pyrazol-6-one	Pyrans	159530635.1
M197T70_pos	197.0809634	70.45495	4-(2-Methyl-6-oxopyran-3-yl)butanoic acid	Pyrans	12073823.3
M253T509_pos	252.9719511	508.639	6-(4-Bromophenyl)-2H-pyran-2-one	Pyrans	21213651
M177T234_pos	177.054651	233.988	Gibepyrone D	Pyrans	34276274.71
M213T282_pos	213.1122411	282.264	Hydroxypestalotin	Pyrans	49563866.44
M125T60_pos	125.0599384	59.5227	Indalone	Pyrans	46668493.84
M150T308_pos	150.0914491	307.989	.alpha.-Cyclopropyl-3-pyridinemethanol	Pyridines_and_derivatives	7894008.463
M304T156_pos	304.2998283	156.031	1-Hexadecylpyridinium cation	Pyridines_and_derivatives	10123357.31
M206T324_pos	206.1287597	324.175	1-Isonicotinoyl-4-piperidinylamine	Pyridines_and_derivatives	5428630.495
M153T333_1_pos	153.0658857	332.571	1-Methyl-4-oxo-1,4-dihydropyridine-3-carboxamide	Pyridines_and_derivatives	14455084.96
M96T65_pos	96.04485385	65.3926	2(1H)-Pyridinone	Pyridines_and_derivatives	140554429
M152T348_pos	152.0706448	348.036	2,4-Dimethylnicotinic acid	Pyridines_and_derivatives	20575125.69
M168T522_pos	168.0292301	522.448	2,4-Pyridinedicarboxylic acid	Pyridines_and_derivatives	5464116.835
M177T379_1_pos	176.9947937	378.888	2,6-Dichloro-4-methyl-3-pyridinamine	Pyridines_and_derivatives	2092866.793
M142T587_pos	142.0350733	587.232	2-(Methylsulfinyl)pyridine	Pyridines_and_derivatives	36081546.36
M167T884_pos	167.0486145	884.243	2-Carbamoylpyridine-3-carboxylic acid	Pyridines_and_derivatives	16056625.41
M154T397_pos	154.0499842	396.762	2-Hydroxy-6-methylpyridine-4-carboxylic acid	Pyridines_and_derivatives	8055724.99
M443T402_pos	443.0605676	402.356	3-Pyridinecarboxamide, 6-[(aminocarbonyl)(2,6-difluorophenyl)amino]-2-(2,4-difluorophenyl)-	Pyridines_and_derivatives	13423073.06
M105T479_pos	105.0426819	479.121	3-cyanopyridine	Pyridines_and_derivatives	66222227.39
M95T421_pos	95.06083353	421.3325	4-Aminopyridine	Pyridines_and_derivatives	6488373.358
M108T291_pos	108.0559863	290.7455	4-Hydrazinylpyridin-2(1H)-one	Pyridines_and_derivatives	6074364.301
M124T433_pos	124.0394904	433.263	4-Pyridinecarboxylic acid	Pyridines_and_derivatives	787275.0936
M184T211_pos	184.0638922	210.716	4-Pyridoxic acid	Pyridines_and_derivatives	13802782.47
M128T271_1_pos	128.0622505	270.78	5-Fluoro-2-hydrazinylpyridine	Pyridines_and_derivatives	15122641.33
M125T104_pos	125.0711703	103.614	6-Methoxy-3-pyridinamine	Pyridines_and_derivatives	129600769.3
M160T352_pos	160.0369023	352.354	6-Methylnicotinic acid	Pyridines_and_derivatives	42656634.43
M254T417_pos	254.1611285	416.925	Anabasamine	Pyridines_and_derivatives	354898.6792
M168T468_pos	168.0291572	468.228	Isocinchomeronic acid	Pyridines_and_derivatives	9546486.307
M381T355_pos	381.1178627	354.988	N'-(4-Methoxybenzoyl)-2-{[4-methyl-5-(3-pyridinyl)-4H-1,2,4-triazol-3-yl]sulfanyl}acetohydrazide	Pyridines_and_derivatives	32160076.06
M167T81_pos	167.0816103	80.7134	N-(2-Hydroxyethyl)pyridine-3-carboxamide	Pyridines_and_derivatives	445051480.3
M264T436_pos	264.1341476	436.393	N2,N4-Bis(2-methoxyethyl)-2,4-pyridinedicarboxamide	Pyridines_and_derivatives	3477813.894
M123T69_1_pos	123.0555141	69.1835	Niacinamide	Pyridines_and_derivatives	232261488.5
M163T240_pos	163.123055	240.322	Nicotine	Pyridines_and_derivatives	22802669.84
M124T405_pos	124.0395329	404.769	Nicotinic acid	Pyridines_and_derivatives	17588053.79
M369T33_pos	369.0968335	32.80365	Nifedipine	Pyridines_and_derivatives	2692801.436
M263T430_pos	262.9120348	430.1	Picloram	Pyridines_and_derivatives	29166640.37
M80T65_pos	80.05002701	65.3926	Pyridine	Pyridines_and_derivatives	15836517.4
M151T44_pos	151.0867219	44.3214	Pyridoxamine	Pyridines_and_derivatives	107232749.5
M253T360_pos	253.0332752	360.488	Thiacloprid	Pyridines_and_derivatives	6273791.028
M193T349_pos	193.0971999	348.637	trans-3'-Hydroxycotinine	Pyridines_and_derivatives	8629236.153
M130T640_pos	130.0864293	639.799	.beta.-Homoproline	Pyrrolidines	4882922.786
M210T310_pos	210.0522478	309.945	1-(2-Chloro-4-nitrophenyl)pyrrolidine	Pyrrolidines	36388462.3
M254T39_2_pos	254.2478268	38.52875	1-Dodecyl-2-pyrrolidinone	Pyrrolidines	51192335.1
M218T328_pos	218.0295577	328.175	2-(2,3-Dichlorophenyl)pyrrolidine	Pyrrolidines	33464929.71
M186T411_pos	186.1125927	410.713	5-Oxo-1-propyl-2-pyrrolidineacetic acid	Pyrrolidines	4883585.227
M142T326_pos	142.0863292	325.721	Ethosuximide	Pyrrolidines	17906422.21
M142T350_pos	142.0863212	350.055	Methyl 1-(2-Methoxyethyl)-2-oxopyrrolidine-4-carboxylate	Pyrrolidines	2668756.028
M101T507_pos	101.0713851	507.026	N-Nitrosopyrrolidine	Pyrrolidines	9764289.238
M72T516_pos	72.08138499	516.072	Pyrrolidine	Pyrrolidines	19627782.06

M296T288_pos	296.042592	288.277	(6-Chloro-2-hydroxy-4-phenyl-3-quinolinyl)acetic acid	Quinolines_and_derivatives	3316242.964
M120T195_pos	120.0810103	195.249	1,2,3,4-Tetrahydroisoquinolin-5-amine	Quinolines_and_derivatives	8411949.495
M519T66_pos	519.3318024	65.5944	2-Heptyl-4-hydroxyquinoline N-oxide	Quinolines_and_derivatives	1447264390
M223T439_pos	222.9882445	438.8395	3-Bromoquinolin-4-amine	Quinolines_and_derivatives	11083716.06
M194T333_pos	194.0449094	332.571	3a,4,5,9b-Tetrahydro-3H-cyclopenta[c]quinoline-4,8-dicarboxylic acid	Quinolines_and_derivatives	8698098.278
M239T348_pos	239.0185538	348.001	4-Chloro-1-methyl-3-nitro-2(1H)-quinolin-2-one	Quinolines_and_derivatives	21719348.35
M332T533_pos	332.1925743	532.541	4-Hydroxy-3-[1-(5-hydroxy-2,6,6-trimethyltetrahydro-2H-pyran-2-yl)ethyl]-2(1H)-quinolinone	Quinolines_and_derivatives	36773080.33
M174T254_pos	174.0550066	253.767	4-quinolinecarboxylate	Quinolines_and_derivatives	38211960.23
M515T421_pos	515.12752	420.757	5-(3-(2-(7-Chloroquinolin-2-yl)ethenyl)phenyl)-8-dimethylcarbamyl-4,6-dithiaoctanoic acid	Quinolines_and_derivatives	11211596.61
M262T448_pos	261.9279291	447.8215	6-Bromo-4-chloro-8-fluoroquinoline	Quinolines_and_derivatives	10876177.9
M224T449_2_pos	223.9721305	449.171	6-Bromoquinolin-2-ol	Quinolines_and_derivatives	10449507.94
M222T578_pos	222.0291676	577.76	7-Chloro-3-methylquinoline-8-carboxylic acid	Quinolines_and_derivatives	13469852.91
M234T509_pos	234.0971148	508.937	7-[Anilino(phenyl)methyl]-8-quinolinol	Quinolines_and_derivatives	2317603.084
M220T195_pos	220.0638939	195.249	8-Methoxy-4-oxo-1,4-dihydroquinoline-2-carboxylic acid	Quinolines_and_derivatives	6104833.49
M266T594_pos	266.1597057	593.58	Acridine Orange	Quinolines_and_derivatives	4752976.567
M394T222_pos	394.120174	221.814	Amsacrine	Quinolines_and_derivatives	47120815.99
M332T402_pos	332.1337534	401.537	Ciprofloxacin	Quinolines_and_derivatives	39239864.38
M200T423_pos	200.0684965	423.396	Dictamine	Quinolines_and_derivatives	44315539.22
M228T241_pos	228.1018828	241.3615	Flindersine	Quinolines_and_derivatives	70835598.03
M393T101_pos	393.2092425	101.485	Indacaterol	Quinolines_and_derivatives	24261304.68
M190T358_pos	190.0499276	358.279	Kynurenic acid	Quinolines_and_derivatives	25323997.78
M180T510_pos	180.076749	509.705	Phenanthridine	Quinolines_and_derivatives	1444680.293
M144T245_pos	144.0444857	245.408	Quinoline-2,8-diol	Quinolines_and_derivatives	1869708.718
M345T311_pos	345.1913203	310.668	Sb 205607	Quinolines_and_derivatives	20435370.23
M206T190_pos	206.0459293	189.537	Xanthurenic acid	Quinolines_and_derivatives	1213569.919
M199T32_pos	199.0754168	32.2006	Diethylstilbestrol	Stilbenes	15075611.09
M257T202_pos	257.1130851	201.734	Pterostilbene	Stilbenes	24801268.74
M517T517_pos	517.1141202	516.837	Salvianolic acid A	Stilbenes	8297419.892
M495T297_pos	495.1493661	297.242	Picroside II	Tannins	66054736.2
M325T402_1_pos	325.0318763	402.356	Dorzolamide	Thiophenes	206968199.1
M309T402_pos	309.0579563	401.537	Dye X-25377-20	Thiophenes	57683441.69
M173T912_pos	173.0855149	911.94	6-Phenyl-1,2,4-triazin-3-amine	Triazines	27067650.64
M216T115_pos	216.1019707	115.376	Atrazine	Triazines	106015653.2
M254T299_pos	254.1386578	299.46	Irgarol	Triazines	19162004.08
M127T227_pos	127.0735541	227.471	Melamine	Triazines	93651376.24

Met ID	m/z	Retention Time(s)	Metabolite Name	Class	A2
M245T414_1_neg	245.0307547	413.518	1-(4-Fluorophenyl)-3-(trifluoromethyl)-1H-pyrazol-5-ol	Azoles	170992659
M84T188_neg	84.0192635	187.825	1h-1,2,3-triazole-5-oh	Azoles	594011.897
M221T396_neg	221.0087102	396.055	3-(2-Chlorophenyl)-1H-pyrazole-5-carboxylic acid	Azoles	296756283
M221T25_neg	221.0086537	25.418	3-(4-Chlorophenyl)-1H-pyrazole-5-carboxylic acid	Azoles	503206930
M156T341_neg	156.0041828	340.589	3-Nitro-1H-pyrazole-5-carboxylic acid	Azoles	243952.716
M158T141_neg	158.0450852	140.531	4-(4-Nitrophenyl)-1H-imidazole	Azoles	2968769.12
M225T511_neg	225.003518	511.305	4-Amino-5-(4-chlorophenyl)-4H-1,2,4-triazole-3-thiol	Azoles	264781557
M126T319_neg	126.0186262	319.335	4-Methyloxazole-5-carboxylic acid	Azoles	459679.514
M265T31_neg	265.0351629	31.3729	4-[3-(4-Chlorophenyl)-1,2,4-oxadiazol-5-yl]butanoic acid	Azoles	624287303
M242T277_neg	242.0349449	277.35	5-(2-Furyl)-4-phenyl-4H-1,2,4-triazol-3-yl hydrosulfide	Azoles	325454.024
M208T199_neg	208.0279223	199.014	5-(4-Fluoro-3-nitrophenyl)-1H-tetraazole	Azoles	4151224.96
M194T58_neg	194.0104803	58.2738	5-(4-Fluorophenyl)-1,3-oxazole-2(3H)-thione	Azoles	7019720.34
M381T388_neg	381.1302684	387.713	5-(4-tert-Butylphenyl)-4-({(E)-[4-(methylsulfanyl)phenyl]methylidene}amino)-2,4-dihydro-3H-1,2,4-triazole-3-thione	Azoles	30910654.9
M165T54_neg	165.0661225	54.4732	5-[2-(1H-Tetraazol-5-yl)ethyl]-1H-tetraazole	Azoles	136759515
M364T295_neg	364.151171	295.388	5-[[5-[2-(3-Aminopropoxy)-6-methoxyphenyl]-1H-pyrazol-3-yl]amino]pyrazinonitrile	Azoles	4308871.89
M157T224_neg	157.0359426	224.146	Allantoin	Azoles	34981180.2
M213T266_neg	213.1239501	266.337	Dethiobiotin	Azoles	16774257
M413T342_2_neg	413.0875441	341.756	Pyrazosulfuron-ethyl	Azoles	22498148
M219T297_neg	219.050462	296.552	[2-(1H-Tetraazol-5-yl)phenoxy]acetic acid	Azoles	61738502.2
M277T120_neg	277.1077696	119.891	1,2-Di(1H-benzimidazol-2-yl)ethanol	Benzimidazoles	38901821.7
M271T206_neg	271.024617	206.079	2-(4-Chlorophenyl)-1H-benzimidazole-5-carboxylic acid	Benzimidazoles	39231492.3
M223T38_neg	223.0260486	37.84245	3-(5-Chloro-1H-benzimidazol-2-yl)propanoic acid	Benzimidazoles	1072567269
M201T243_neg	201.0397698	242.984	5-Nitro-2-(1,3-thiazol-4-yl)-1H-1,3-benzodiazole	Benzimidazoles	182613304
M239T58_neg	239.0556418	57.7149	5-Nitro-2-(2-pyridinyl)-1H-benzimidazole	Benzimidazoles	896789412
M261T50_2_neg	261.0765874	49.5621	N-(1H-Benzimidazol-2-yl)-3-cyanobenzamide	Benzimidazoles	85604168.3
M281T182_neg	281.066249	182.217	N-(1H-Benzimidazol-2-yl)-3-nitrobenzamide	Benzimidazoles	78561825.3
M273T110_neg	273.0349716	109.518	Phenylbenzimidazolesulfonic acid	Benzimidazoles	39676815.7
M393T456_neg	392.9508361	456.311	Selumetinib	Benzimidazoles	1473868.08
M271T276_neg	271.0590079	276.2975	3-(2,6-Dihydroxyphenyl)-4-hydroxy-6-methyl-3H-2-benzofuran-1-one	Benzofurans	74702822.4
M163T283_neg	163.0028261	283.268	3-Hydroxyphthalic anhydride	Benzofurans	89430810.8
M179T456_neg	179.0190265	456.43	5-Fluoro-1-benzofuran-2-carboxylic acid	Benzofurans	3539484.52
M252T418_neg	252.0622763	418.305	Furegrelate	Benzofurans	1549948.26
M209T277_neg	209.008616	276.892	Trimellitic acid anhydride	Benzofurans	334373581
M345T28_neg	345.0611763	27.5835	(1S)-1,5-Anhydro-1-(1,3,6-trihydroxy-5-methoxy-9-oxo-9H-xanthen-2-yl)-D-glucitol	Benzopyrans	195976954
M261T350_neg	261.040142	350.107	1,8-Dihydroxy-9-oxo-2,3-dihydro-1H-cyclopenta[b]chromene-6-carboxylic acid	Benzopyrans	37052799.7
M205T227_neg	205.0499393	226.899	3-(5,7-Dimethoxy-4-oxochromen-2-yl)propanoic acid	Benzopyrans	36363297.5
M233T30_neg	233.0451182	30.2902	Anhydrobrazilic acid	Benzopyrans	335222850
M273T53_neg	273.0402891	53.10675	Anomalin A	Benzopyrans	48080035.5
M163T112_neg	163.0391977	111.857	Brazilin	Benzopyrans	405376065
M285T241_neg	285.0402855	240.722	Calyxanthone	Benzopyrans	145667184
M189T374_neg	189.0187026	374.04	Chromone-2-carboxylic acid	Benzopyrans	450831592
M249T38_neg	249.0769772	37.7594	Citrinin	Benzopyrans	532379984
M303T324_neg	303.0507458	324.478	Corymbiferin	Benzopyrans	61563109.1
M435T282_neg	435.0927912	282.49	Irisxanthone	Benzopyrans	270077925
M407T60_neg	407.0979613	60.4391	Khelloside	Benzopyrans	95159954.9
M351T25_neg	351.0870539	25.418	Mangostine	Benzopyrans	159718044
M431T364_neg	431.0826812	364.483	Naphthofluorescein	Benzopyrans	58279256.3
M257T29_neg	257.0452266	29.2073	Norlichexanthone	Benzopyrans	330995933
M381T387_neg	381.0825635	387.442	Resorcinolnaphthalein	Benzopyrans	34587064.9
M115T56_neg	115.0026914	55.87585	Fumarate	Carboxylic_acids_and_derivatives	6277541919
M117T432_3_neg	117.0183706	432.255	Succinate	Carboxylic_acids_and_derivatives	3003033793
M295T36_neg	295.0819724	36.2275	Penicillamine disulfide	Carboxylic_acids_and_derivatives	972858229
M103T435_2_neg	103.0026647	434.546	Malonic acid	Carboxylic_acids_and_derivatives	879273338
M309T26_2_neg	309.1739781	25.81845	Gly-Pro-Arg-Pro-amide	Carboxylic_acids_and_derivatives	811959706
M173T400_2_neg	173.008545	399.653	Isocitric acid	Carboxylic_acids_and_derivatives	740701015
M191T504_4_neg	191.0190949	503.836	Citric acid	Carboxylic_acids_and_derivatives	615566797
M353T26_neg	353.2001181	26.45255	(E)-3-(4-Acetyloxy-2,3-dihydroxy-2,5,5,8a-tetramethyl-3,4,4a,6,7,8-hexahydro-1H-naphthalen-1-yl)prop-2-enoic acid	Carboxylic_acids_and_derivatives	497725162
M73T63_neg	72.99203299	63.0779	Glyoxylate	Carboxylic_acids_and_derivatives	384250238
M271T250_neg	271.0820915	250.3725	.gamma.-Glutamyl-(S)-allyl-L-cysteine	Carboxylic_acids_and_derivatives	356782287
M129T243_1_neg	129.0183331	242.527	trans-Glutaconic acid	Carboxylic_acids_and_derivatives	329963959
M128T354_3_neg	128.0343433	353.5345	L-pyroglutamic acid	Carboxylic_acids_and_derivatives	326341842
M231T30_neg	231.0658094	29.754	7-(Difluoromethyl)-5-methyl-4,5,6,7-tetrahydro[1,2,4]triazolo[1,5-a]pyrimidine-2-carboxylic acid	Carboxylic_acids_and_derivatives	324376243
M281T76_neg	281.0663187	75.9665	5-(2,5-Dioxooxolan-3-yl)-7-methyl-1,3,3a,4,5,7a-hexahydro-2-benzofuran-1,3-dione	Carboxylic_acids_and_derivatives	278153188
M113T244_neg	113.0233718	243.927	Glutaric acid	Carboxylic_acids_and_derivatives	264753552
M189T410_neg	189.0033693	410.2375	(-)-Hydroxycitric acid lactone	Carboxylic_acids_and_derivatives	262107099
M267T105_neg	267.0507356	104.773	L-Homocystine	Carboxylic_acids_and_derivatives	239140674
M177T52_2_neg	177.0550347	52.2469	(3E)-4-(3,4-Dihydroxyphenyl)-3-buten-2-one	Cinnamic_acids_and_derivatives	2.2648E+10
M471T515_neg	471.1350956	514.663	1,6-Bis-O-(4-hydroxycinnamoyl)glucose	Cinnamic_acids_and_derivatives	212290787
M383T27_neg	383.0768979	26.5006	3,4-(Methylenedioxy)cinnamic acid	Cinnamic_acids_and_derivatives	207990271
M273T430_neg	273.0403509	429.747	3-Hydroxy-2-[(E)-3-(4-hydroxyphenyl)prop-2-enoyl]oxy-3-methoxycarbonylpentanedioic acid	Cinnamic_acids_and_derivatives	456790838
M187T355_neg	187.0392935	355.402	3-O-Acetyl-2-O-((2E)-3-(4-hydroxyphenyl)prop-2-enoyl)hexopyranose	Cinnamic_acids_and_derivatives	24564771.4
M191T31_neg	191.034321	30.82515	4-(2-Carboxyethenyl)benzoic acid	Cinnamic_acids_and_derivatives	176289264
M149T361_neg	149.0446417	361.002	4-[(2E)-3-(3,4-Dihydroxyphenyl)prop-2-enoyl]oxy-2,3-dihydroxy-2-methylbutanoic acid	Cinnamic_acids_and_derivatives	106678713
M519T436_neg	519.1464449	436.081	6-Hydroxy-1-((2-O-((2E)-3-(4-hydroxyphenyl)prop-2-enoyl)hexopyranosyl)oxy)-7-methylidene-1,4a,5,6,7,7a-hexahydrocyclopenta[c]pyran-4-carboxylic acid	Cinnamic_acids_and_derivatives	22716465.1
M317T414_neg	317.0545616	413.722	Belinostat	Cinnamic_acids_and_derivatives	9285388.63
M135T455_neg	135.0442193	454.954	Caffeate	Cinnamic_acids_and_derivatives	87938222.1
M311T291_1_neg	311.0405504	290.871	Caftaric acid	Cinnamic_acids_and_derivatives	199783881
M477T256_neg	477.1397123	256.218	Calceolarioside A	Cinnamic_acids_and_derivatives	157388243
M191T111_neg	191.0726797	111.042	Dehydrozingerone	Cinnamic_acids_and_derivatives	71748965.3
M439T249_neg	439.1970136	249.406	Diferuloyl putrescine	Cinnamic_acids_and_derivatives	2594448.9
M265T238_neg	265.0714976	238.301	NCGC00385221-01	Cinnamic_acids_and_derivatives	113436705
M593T327_neg	593.1509302	326.942	Safflor yellow A	Cinnamic_acids_and_derivatives	56273418.5
M175T26_neg	175.0390621	25.9542	Sibiricose A5	Cinnamic_acids_and_derivatives	258991039
M223T232_1_neg	223.0606535	232.113	Sinapic acid	Cinnamic_acids_and_derivatives	169005191
M163T34_neg	163.0392295	34.0667	p-Coumaric acid	Cinnamic_acids_and_derivatives	452435049
M163T238_neg	163.0391774	238.301	trans-2-Hydroxycinnamic acid	Cinnamic_acids_and_derivatives	66549904.6
M193T101_neg	193.0499151	100.816	trans-Ferulic acid	Cinnamic_acids_and_derivatives	538892141
M203T39_neg	203.0343045	38.9153	3-Carboxy-6-methylcoumarin	Coumarins_and_derivatives	135897047
M161T25_neg	161.0230215	25.418	3-Chloro-5,7-dihydroxy-4-methylcoumarin	Coumarins_and_derivatives	58285046.3
M145T39_neg	145.0285417	39.4618	3-Fluoro-7-hydroxy-4-methylcoumarin	Coumarins_and_derivatives	131100992
M351T101_neg	351.0717572	101.393	4-Methylumbelliferyl .beta.-D-glucuronide	Coumarins_and_derivatives	218720573
M191T362_1_neg	191.0342672	362.365	5,7-Dihydroxy-4-methylcoumarin	Coumarins_and_derivatives	46856320.1
M235T283_2_neg	235.0243747	283.268	6,7-Dihydroxycoumarin-4-acetic acid	Coumarins_and_derivatives	210825258
M211T136_neg	211.0242087	136.458	6,8-Difluoro-7-hydroxy-4-methylcumarin	Coumarins_and_derivatives	609262623
M191T269_neg	191.0342797	269.488	6-Hydroxy-7-methoxycoumarin	Coumarins_and_derivatives	376190571
M117T314_neg	117.033648	313.796	6-Hydroxycoumarin	Coumarins_and_derivatives	991072957
M191T60_neg	191.0343015	60.4391	7,8-Dihydroxy-4-methylcoumarin	Coumarins_and_derivatives	301142835
M249T289_neg	249.0400571	289.305	7,8-Dihydroxy-4-methylcoumarin-3-acetic acid	Coumarins_and_derivatives	185335927
M257T367_neg	257.0299697	367.195	7-Hydroxy-4-methyl-3-(2-thiophenyl)coumarin	Coumarins_and_derivatives	66656297.2
M147T120_neg	147.0442309	119.891	7-Hydroxy-4-methylchromen-2-one	Coumarins_and_derivatives	100859416
M205T314_neg	205.0136854	313.796	7-Hydroxycoumarin-3-carboxylic acid	Coumarins_and_derivatives	654164410
M219T362_1_neg	219.029374	362.365	7-Hydroxycoumarin-4-acetic acid	Coumarins_and_derivatives	123545716
M293T206_neg	293.1062113	206.219	7-methoxy-6-(1,2,3-trihydroxy-3-methylbutyl)chromen-2-one	Coumarins_and_derivatives	21666892.2
M257T242_neg	257.0453113	242.071	Alternariol	Coumarins_and_derivatives	707672737
M161T314_2_neg	161.0237277	313.796	Coumachlor	Coumarins_and_derivatives	2388004202
M339T405_neg	339.0718142	405.159	Daphnin	Coumarins_and_derivatives	35186005.9
M177T396_neg	177.0185114	396.055	Esculin	Coumarins_and_derivatives	115282330
M205T364_neg	205.0136568	364.483	Ethyl 7-hydroxy-2-oxo-2H-chromene-3-carboxylate	Coumarins_and_derivatives	25828196.4
M429T341_neg	429.1031986	340.945	Fraxidinglucoside	Coumarins_and_derivatives	110669468
M369T303_neg	369.0824077	303.259	Fraxin	Coumarins_and_derivatives	20715197.8
M221T50_neg	221.0450606	49.5621	Isofraxidin	Coumarins_and_derivatives	726733861
M249T347_neg	249.0400804	346.6785	Methyl 6,7-dihydroxycoumarin-4-acetate	Coumarins_and_derivatives	32698232.1
M191T283_neg	191.0343321	283.268	Scopoletin	Coumarins_and_derivatives	625947605
M191T25_neg	190.9979128	24.8717	Umckalin	Coumarins_and_derivatives	98052306.1
M171T97_2_neg	171.1019256	96.875	(2E)-4-Hydroxynon-2-enoic acid	Hydroxy_acids_and_derivatives	673281168
M281T223_2_neg	281.1391332	222.9	(7E)-4-Hydroxy-7-(1-hydroxypropan-2-ylidene)octahydro-3,9a-methanocyclopenta[c]oxocine-4(1H)-carboxylic acid	Hydroxy_acids_and_derivatives	73801743.5
M103T257_neg	103.0389901	256.91	.alpha.-Hydroxybutyric acid	Hydroxy_acids_and_derivatives	14823104.1
M103T243_neg	103.0389859	242.527	.alpha.-Hydroxyisobutyric acid	Hydroxy_acids_and_derivatives	72218506.1
M89T338_neg	89.02337739	338.264	.beta.-hydroxypropionic acid	Hydroxy_acids_and_derivatives	425658694
M187T119_2_neg	187.1332918	118.72	10-hydroxydecanoate	Hydroxy_acids_and_derivatives	18146940.5
M229T369_neg	229.1075728	369.163	2-(6-Hydroxyhexyl)-3-methylidenebutanedioic acid	Hydroxy_acids_and_derivatives	88798917.3
M143T481_neg	142.9952504	480.741	3,3,3-Trifluorolactic acid	Hydroxy_acids_and_derivatives	108809707
M171T50_neg	171.0291039	50.0882	3-Dehydroquinic acid	Hydroxy_acids_and_derivatives	388433509
M103T278_neg	103.0390052	278.232	3-Hydroxybutyric acid	Hydroxy_acids_and_derivatives	53986795.3
M375T36_neg	375.2750197	36.3619	3-Hydroxycapric acid	Hydroxy_acids_and_derivatives	4836235.87
M147T304_neg	147.0290762	304.163	3-Hydroxyglutaric acid	Hydroxy_acids_and_derivatives	8880354.55
M133T445_2_neg	133.0133862	444.876	DL-Malic acid	Hydroxy_acids_and_derivatives	1290514151
M203T204_neg	203.128239	203.89	Decanoic acid, 3,5-dihydroxy-	Hydroxy_acids_and_derivatives	11299168.9
M89T266_1_neg	89.02339494	265.6865	Dl-lactate	Hydroxy_acids_and_derivatives	2666765301
M173T124_neg	173.117523	124.362	Fa 9:0+1o	Hydroxy_acids_and_derivatives	46960270.9

M195T425_neg	195.0505825	424.774	Galactonic acid	Hydroxy_acids_and_derivatives	469790849
M75T321_neg	75.00772361	320.6165	Glycolate	Hydroxy_acids_and_derivatives	1781134350
M217T382_neg	217.1076407	382.3765	Hydroxysebacic acid	Hydroxy_acids_and_derivatives	53871948.7
M147T435_2_neg	147.0290903	434.924	L-2-Hydroxyglutaric acid	Hydroxy_acids_and_derivatives	282879143
M133T588_neg	133.0132813	587.588	Malate	Hydroxy_acids_and_derivatives	31561623.1
M209T101_neg	209.0675271	100.698	(3Z)-3-(1H-Pyrrol-2-ylmethylene)-1,3-dihydro-2H-indol-2-one	Indoles_and_derivatives	39306611
M177T246_neg	177.0397493	246.094	(3Z)-6-Hydroxy-3-(1H-imidazol-5-ylmethylidene)-12-methoxy-7a-(2-methylbut-3-en-2-yl)-7a,12-dihydro-1H,5H-imidazo[1',2':1,2]pyrido[2,3-b]indole-2,5(3H)-dione	Indoles_and_derivatives	46067714
M188T280_neg	188.0557254	279.952	1,3,4,5-Tetrahydrothiopyrano[4,3-b]indole	Indoles_and_derivatives	11608429.8
M303T281_neg	303.1272674	281.414	1H-Indole-1-acetic acid, 5-fluoro-2-methyl-3-(2-quinolinylmethyl)-	Indoles_and_derivatives	3911149.17
M384T414_neg	384.1595577	414.378	1H-Indole-1-pentanoic acid, 3-[(4-methyl-1-naphthalenyl)carbonyl]-	Indoles_and_derivatives	13012835.4
M323T359_neg	323.0768536	358.585	1H-Indole-2,3-dione, 5-[[2-(methoxymethyl)-1-pyrrolidinyl]sulfonyl]-	Indoles_and_derivatives	68429383.1
M160T93_neg	160.0395328	93.2153	1H-Indole-3-carboxylic acid	Indoles_and_derivatives	76751302.3
M248T348_neg	248.1136174	348.119	1H-Indole-3-carboxylic acid, 1-(5-fluoropentyl)-	Indoles_and_derivatives	1340224.47
M276T431_neg	276.1086224	431.341	2-[(4-Hydroxyanilino)methyl]-1-methyl-1H-indole-3-carbonitrile	Indoles_and_derivatives	5275026.91
M142T324_neg	142.0652927	323.858	3-Indoleacrylic acid	Indoles_and_derivatives	1040376.69
M321T46_neg	321.0612143	46.1781	3-Pyridinecarboxamide, N-(6-chloro-9H-pyrido[3,4-b]indol-8-yl)-	Indoles_and_derivatives	94505374.8
M219T342_neg	219.076985	341.8385	5-Hydroxy-L-tryptophan	Indoles_and_derivatives	14068933.3
M232T66_neg	232.0281025	65.5887	7-(Trifluoromethyl)-1H-indole-2,3-dione	Indoles_and_derivatives	10877731
M237T29_neg	237.0418141	29.25625	7-Chloro-L-tryptophan	Indoles_and_derivatives	785053262
M159T375_3_neg	159.0766536	375.381	Ala-Ala	Indoles_and_derivatives	4501935.41
M220T382_neg	220.0822563	382.499	Ethyl 5-fluoro-3-methyl-1H-indole-2-carboxylate	Indoles_and_derivatives	896575.98
M144T38_neg	144.0445726	37.8008	Indole-3-carboxaldehyde	Indoles_and_derivatives	18499325.6
M312T367_neg	312.0734437	366.895	Indomethacin	Indoles_and_derivatives	339966.977
M146T265_neg	146.0601879	264.686	Isopropyl 5-hydroxy-2-methyl-1H-indole-3-carboxylate	Indoles_and_derivatives	2297764.06
M219T362_2_neg	219.0450677	361.52	Methyl 6-nitro-1H-indole-2-carboxylate	Indoles_and_derivatives	161071835
M397T127_neg	397.3319687	126.739	N-Palmitoyltryptamine	Indoles_and_derivatives	253193077
M181T307_neg	181.0974611	307.26	Protubonine A	Indoles_and_derivatives	12960719.5
M271T435_neg	271.1297854	434.608	Sempervirine	Indoles_and_derivatives	512620.926
M203T321_neg	203.082083	321.413	Tryptophan	Indoles_and_derivatives	1310784.83
M473T237_neg	473.2541694	236.679	Amastatin	Peptidomimetics	50364554.7
M512T524_neg	512.2181834	524.113	Astin B	Peptidomimetics	6497481.76
M153T390_neg	153.0547909	389.549	2-(2-Hydroxyethoxy)phenol	Phenol_ethers	17672946.2
M369T49_neg	369.0975224	49.0259	Mitoglitazone	Phenol_ethers	442001589
M355T410_neg	355.1048284	409.943	Pioglitazone	Phenol_ethers	61116269.6
M150T145_neg	150.0187329	145.226	2,3,4-Trihydroxybenzaldehyde (4-chlorophenyl)hydrazone	Phenols	271484895
M251T48_neg	251.0193673	48.4893	2,4-Dinitro-6-(1H-tetrazol-1-yl)phenol	Phenols	71303870.7
M183T27_neg	183.0041177	26.9509	2,4-dinitrophenol	Phenols	49106600.5
M219T473_neg	219.0982026	472.558	2-Hydroxy-4-methoxy-5-(2-methylbut-3-en-2-yl)benzaldehyde	Phenols	382809.04
M165T32_1_neg	165.0185846	32.3873	3,4-Dihydroxymandelic acid	Phenols	1.3445E+10
M123T60_neg	123.0441215	60.4391	3,4-Dihydroxyphenylacetic acid	Phenols	118095604
M139T243_neg	139.0391277	242.527	3,5-Dihydroxybenzenemethanol	Phenols	100127473
M139T30_neg	139.003443	30.1149	3,5-Dinitrocatechol	Phenols	369555323
M121T416_neg	121.0285262	416.395	3-Chloro-5-methylbenzene-1,2-diol	Phenols	102592156
M277T913_neg	276.9313294	913.0315	3-Hydroxy-2-iodo-4-methoxybenzaldehyde	Phenols	27536669.9
M151T416_neg	151.0392329	416.395	3-Hydroxy-2-methoxybenzaldehyde	Phenols	25321847.5
M107T369_neg	107.0491688	368.552	3-Hydroxyphenylacetic acid	Phenols	67401401
M387T278_neg	387.0718644	278.4265	3-Isoxazolecarboxamide, 5-(5-chloro-2,4-dihydroxyphenyl)-N-ethyl-4-(4-methoxyphenyl)-	Phenols	46516418
M151T393_neg	151.0392605	392.626	4-Hydroxyphenylacetic acid	Phenols	63145312
M123T41_neg	123.0441304	40.535	4-Methylcatechol	Phenols	118125626
M251T237_neg	251.0557989	237.231	4-[2-(2-Pyrimidinylamino)-1,3-thiazol-4-yl]-1,2-benzenediol	Phenols	34638787.6
M331T523_neg	331.0303147	523.132	4-[2-(3-Chloro-4-methylanilino)-1,3-thiazol-4-yl]-1,2-benzenediol	Phenols	14534845.3
M317T518_neg	317.0148338	518.4965	4-[2-(3-Chloroanilino)-1,3-thiazol-4-yl]-1,2-benzenediol	Phenols	980848.184
M167T897_neg	166.9829305	897.352	5-Hydroxy-1,3-benzoxathiol-2-one	Phenols	37746928.5
M439T36_neg	439.2852483	35.6592	5-[(Z)-16-(3,5-Dihydroxyphenyl)hexadec-12-enyl]benzene-1,3-diol	Phenols	18670741.8
M179T261_neg	179.0342897	260.575	DL-4-Hydroxy-3-methoxymandelic acid	Phenols	213439805
M297T356_neg	297.0974949	356.113	Moupinamide	Phenols	25198426.4
M109T28_neg	109.0284731	27.7131	Pyrocatechol	Phenols	288775839
M151T476_neg	151.0394643	476.108	Vanillin	Phenols	19030649
M165T39_neg	165.054933	38.76265	Vanylglycol	Phenols	649670515
M321T25_neg	321.2101846	25.3928	[8]-Gingerol	Phenols	34523946.2
M303T31_neg	303.1996493	30.7674	[8]-Shogaol	Phenols	21308759.6
M207T81_neg	207.0655178	80.9079	trans-3,5-Dimethoxy-4-hydroxycinnamaldehyde	Phenols	175805661
M123T100_neg	123.0077542	100.234	(-)-trans-4-[4-(4'-Fluorophenyl)-3-piperidinylmethoxy]-2-methoxyphenol	Piperidines	205875980
M455T301_neg	455.1600736	301.32	1-Piperidinecarboxamide, N-3-pyridinyl-4-[[3-[[5-(trifluoromethyl)-2-pyridinyl]oxy]phenyl]methyl]-	Piperidines	1164329.97
M135T109_neg	135.0441885	108.694	2-(1,2-Dihydroxy-2-phenylethyl)-3-hydroxy-2,3-dihydropyran-6-one	Pyrans	305880407
M125T385_neg	125.0234427	385.216	4-Hydroxy-3-[(4-hydroxy-6-methyl-2-oxopyran-3-yl)methyl]-6-methylpyran-2-one	Pyrans	170399222
M183T37_neg	182.9927448	37.30275	Chelidonic acid	Pyrans	73117666.9
M141T72_neg	141.0180835	72.1485	Kojic acid	Pyrans	1175407110
M166T193_neg	166.017207	192.59	2,3-Pyridinedicarboxylic acid	Pyridines_and_derivatives	34380964.8
M138T414_neg	138.0187025	414.378	3-Hydroxypicolinic acid	Pyridines_and_derivatives	73058910.5
M321T410_neg	321.0248559	409.928	4-(4-Chlorophenyl)-6-phenyl-2-thioxo-1,2-dihydro-3-pyridinecarbonitrile	Pyridines_and_derivatives	39415128.3
M154T60_neg	154.0500516	60.3413	Cycloheximide	Pyridines_and_derivatives	160251940
M122T265_neg	122.023793	264.686	Picolinic acid	Pyridines_and_derivatives	12854535.1
M495T494_2_neg	495.1118151	494.211	Pyridoxamine 5-phosphate	Pyridines_and_derivatives	1047767.83
M208T269_neg	208.0608869	268.843	1-(2-Furanylmethyl)-5-oxo-3-pyrrolidinecarboxylic acid	Pyrrolidines	424797.394
M254T232_neg	254.1031052	231.602	2-(2-Naphthyl)-4-quinolinecarboxylic acid	Quinolines_and_derivatives	7509191.03
M242T86_neg	242.1507459	85.8493	2-Heptylquinolin-4(1H)-one	Quinolines_and_derivatives	38905922.9
M275T260_neg	275.1035406	259.672	Ofloxacin	Quinolines_and_derivatives	35764180.7
M382T432_1_neg	382.1624925	432.15	Peniprequinolone	Quinolines_and_derivatives	1159671.91
M225T299_neg	225.0974778	298.792	4-O-Methylpinosylvic acid	Stilbenes	36994468.5
M201T29_neg	201.0550488	29.4735	Piceatannol	Stilbenes	62472128.6
M403T122_1_neg	403.1030011	121.603	Resveratrol 4'-O-D-glucuronide	Stilbenes	108087183
M583T375_neg	583.1667358	375.37	4-Hydroxyphenyl 2-O-((2S,3R,4S)-3,4-dihydroxy-4-(((4-hydroxy-3,5-dimethoxybenzoyl)oxy)methyl)tetrahydrofuran-2-yl)-.beta.-D-glucopyranoside	Tannins	20003150.5
M255T50_1_neg	255.0506711	49.5621	3-(5-phenylthiophen-2-yl)prop-2-ynyl acetate	Thiophenes	66579769.5
M169T504_neg	168.9985068	504.2655	5-Acetyl-2-thiophenecarboxylic acid	Thiophenes	36936835.6
M563T342_neg	563.1404771	341.756	Dye X-16864-164-B	Thiophenes	19378026.6
M107T52_neg	106.9797308	51.9063	Taurosteine	Thiophenes	43444244.1
